# Fluorinated Hypercrosslinked Polymers with Exceptional Capacity for Perfluorooctanoic Acid Removal from Water

**DOI:** 10.1002/smll.202509398

**Published:** 2026-06-04

**Authors:** Mingqing Yu, Anna Fabisikova, Paul Schweng, Martin Zehl, Huajie Yu, Yaozu Liao, Robert T. Woodward

**Affiliations:** ^1^ State Key Laboratory of Advanced Fiber Materials College of Materials Science and Engineering Donghua University Shanghai China; ^2^ Institute of Materials Chemistry and Research Faculty of Chemistry University of Vienna Vienna Austria; ^3^ Department of Analytical Chemistry Faculty of Chemistry University of Vienna Vienna Austria

**Keywords:** adsorption, hypercrosslinked polymers, noncovalent F‐F interactions, PFAS, PFOA, water purification

## Abstract

Perfluorooctanoic acid (PFOA) is a persistent emerging pollutant that has raised significant environmental and health concerns due to its toxicity, bioaccumulation, and resistance to degradation. The effective removal of PFOA from aqueous systems necessitates the development of advanced adsorbent materials with superior efficiency and capacity. We report the synthesis and characterization of fluorinated hypercrosslinked polymers (FHCPs), which exhibit exceptional adsorption of PFOA from water, including tap and lake samples. Among these, FHCP‐3 achieved an outstanding equilibrium adsorption capacity of 1550 ± 60 mg g^−1^ for PFOA and demonstrated impressive kinetics, reaching equilibrium within 60 min even at low pollutant concentrations (1 mg L^−1^). Notably, FHCP‐3 also displayed facile regeneration and reusability, retaining almost quantitative removal efficiency over six consecutive adsorption–desorption cycles. Density functional theory calculations, including binding energy, Gibbs free energy, and reduced density gradient analysis, further revealed the strong non‐covalent interactions driving selective PFOA adsorption. This work highlights FHCP‐3 as a high‐performing adsorbent that surpasses many state‐of‐the‐art materials in PFOA adsorption capacity, kinetics, and durability while offering a simple, low‐cost synthesis. These findings position FHCP‐3 as a promising platform for the rapid and efficient removal of PFOA, addressing critical challenges in water purification and environmental remediation.

## Graphical abstract

Fluorinated hypercrosslinked polymers (FHCPs) are reported as highly efficient sorbents for the rapid removal of perfluorooctanoic acid (PFOA) from water. The materials integrate strong fluorophilic interactions, extensive porosity, and excellent chemical stability, delivering fast adsorption kinetics, high uptake capacity, and selective PFOA removal under low‐concentration aqueous conditions, offering a promising platform for practical PFAS remediation.

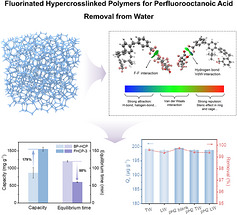

## 1 Introduction

Per‐ and polyfluoroalkyl substances (PFAS) are synthetic chemicals characterized by their unique molecular structure, commonly combining hydrophobic fluorocarbon chains with hydrophilic head groups [1]. The exceptional stability of PFAS arises from the strength of the carbon‐fluorine (C─F) bond, which is among the strongest covalent bonds known to organic chemistry [2]. This remarkable bond strength underpins the thermal and chemical resilience of these compounds, enabling their use in diverse applications across various industrial and commercial domains, such as non‐stick coatings, aqueous firefighting foams, waterproof textiles, and wire coatings [3]. However, the disposal of PFAS‐containing products, combined with the inherent persistence of PFAS molecules, has resulted in their detection in surface water, tap water, and even drinking water [4, 5]. Often referred to as “forever chemicals”, PFAS are recognized for their bioaccumulative propensity in both the environment and the human body [6]. Among PFAS, perfluorooctanoic acid (PFOA) is particularly concerning due to its high‐water solubility and classification by the International Agency for Research on Cancer (IARC) as being potentially carcinogenic to humans, with exposure linked to testicular and kidney cancer [7]. Therefore, there is an urgent need for effective and efficient remediation of PFOA‐contaminated water sources to mitigate its significant threats to human health and the environment.

Efforts to mitigate PFOA contamination in water have focused on various strategies, including adsorption, photocatalysis, electrochemical oxidation, and bioremediation [8]. Adsorption‐based technologies are among the most employed methods for the treatment of PFOA‐contaminated water, encompassing dispersion forces, induction, and electrostatic interactions, or their combinations, for both targeted and non‐targeted applications [9]. Adsorbents such as activated carbon and ion exchange resins are widely utilized in industrial applications. While granular activated carbon (GAC) and powdered activated carbon are cost‐effective options, their efficiency in PFOA removal is relatively low. Conventional GAC exhibits limited adsorption capacity (112–161 mg g^−1^) and slow kinetics, often requiring more than 24 h to reach equilibrium. Ion exchange resins offer similar adsorption capacities ranging from 40 to 170 mg g^−1^, and typically demand even longer equilibration times (50–168 h) [10, 11, 12]. The implementation of these conventional adsorbents is often hindered by limited adsorption efficiency and poor regeneration, rendering them unsuitable for long‐term applications. Recent advances in PFOA remediation have increasingly focused on porous materials such as metal–organic frameworks (MOFs) [13, 14], covalent organic frameworks (COFs) [15, 16], and porous aromatic frameworks (PAFs) [17]. Although many of these systems exhibit high adsorption capacities, their practical deployment remains constrained by high synthesis costs, complex preparation protocols, or limited chemical stability in acidic or alkaline environments. These limitations highlight the need for robust, low‐cost, and scalable adsorbents capable of maintaining high performance in complex matrices, such as natural bodies of water.

Hypercrosslinked polymers (HCPs) are promising adsorbents due to their low‐cost monomers, potential for metal‐free polymerization, and excellent chemical and thermal stability. HCPs are a unique subclass of porous organic polymers characterized by densely interconnected micro‐/mesoporous networks formed via Friedel‐Crafts reactions. The diverse synthetic routes to HCPs, as well as their ease of functionalization, high specific surface areas, low‐cost reagents, and mild reaction conditions, have driven rapid advancements in their development [18]. Their synthesis can proceed via self‐condensation [19] or by “knitting” aromatic compounds using external crosslinking, meaning monomers do not require specifically polymerizable moieties [20]. Unlike many other porous organic polymers, the synthesis of HCPs does not require expensive noble metal catalysts, relying instead on Lewis acids such as ferric chloride or aluminum chloride, significantly reducing production costs and offering inherent scalability advantages. Owing to their facile preparation and adjustable chemical properties, HCPs have demonstrated potential in diverse fields such as gas separation and storage [21], energy storage [22], atmospheric water harvesting [23], and heterogeneous catalysis [24]. Despite these advantages, their application in PFOA remediation remains largely unexplored.

Fluorinated materials possess strong hydrophobicity, chemical resistance, thermal stability, and tunable electronic environments, which make them particularly suitable for interacting with perfluoroalkyl chains [25, 26, 27]. Recent advances in fluorinated COFs and MOFs have revealed that fluorophilic contacts, hydrophobic effects, and local polar interactions play important roles in enhancing PFOA adsorption [28]. Despite these promising properties, such COF‐ and MOF‐based systems often contain coordination or imine linkages that are susceptible to chemical degradation under acidic or alkaline water conditions, limiting their practical use [29]. Embedding fluorine directly into HCP frameworks offers a robust alternative because the C─C backbone provides excellent chemical and thermal stability, while the fluorinated domains may promote selective affinity toward perfluoroalkyl contaminants. Although fluorinated hypercrosslinked polymers (FHCPs) have been studied for gas‐phase adsorption [30], their application in removing PFOA from water has, to the best of our knowledge, not yet been investigated. The development of FHCPs that combine scalability, high stability, large surface area, and intrinsic fluorophilicity would therefore represent an important advancement.

**FIGURE 1:**
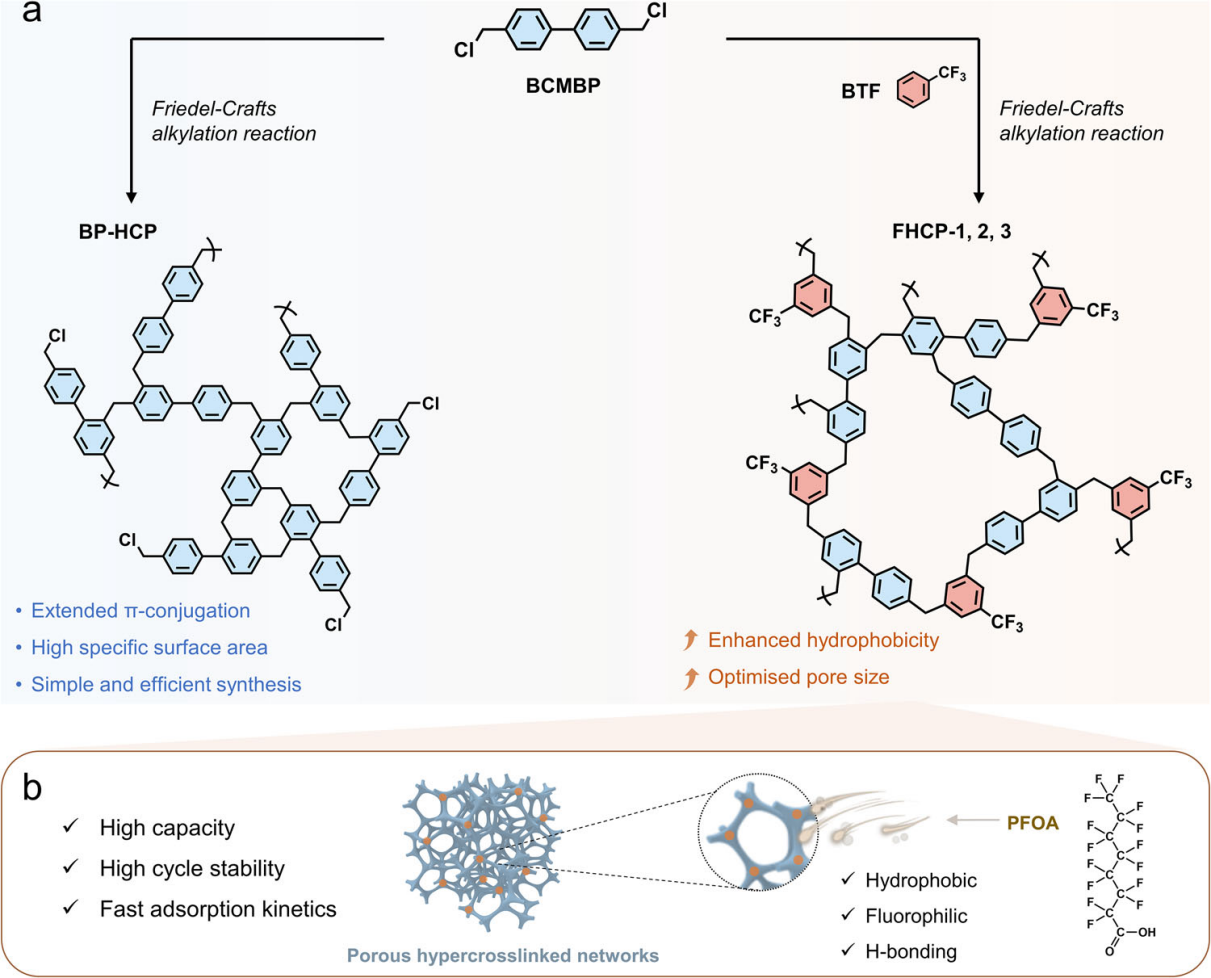
Schematic illustration of (a) the synthesis of BP‐HCP and FHCPs via Friedel‐Crafts alkylation, and (b) the conceptual framework linking fluorinated pore architectures to enhanced PFOA adsorption through hydrophobic, fluorophilic, and hydrogen bonding interactions.

In this work, we report a straightforward Friedel‐Crafts strategy for constructing FHCPs using 4,4'‐bis(chloromethyl)‐1,1'‐biphenyl (BCMBP) and α,α,α‐trifluorotoluene (BTF), yielding materials with high surface areas (up to 1740 ± 61 m^2^ g^−1^), permanent porosity, and tunable fluorine contents. The resulting FHCPs exhibit excellent stability and high adsorption capacity toward PFOA. To gain mechanistic insights into the origin of this high affinity, we investigated the driving forces underlying the adsorption process through density functional theory (DFT) modeling, including binding energy, Gibbs free energy, and non‐covalent interaction analysis. Our FHCPs broaden the range of adsorbent materials for PFAS capture and open new possibilities for advanced wastewater treatment. A schematic overview of the synthetic strategy toward FHCPs and their PFOA adsorption mechanism is presented in Figure 1, highlighting the comparison between the fluorinated FHCPs and a non‐fluorinated counterpart (BP‐HCP). Overall, this study establishes FHCPs as a new class of stable, fluorine‐rich porous organic polymers for high‐performance PFOA adsorption, supported by mechanistic insights from DFT calculations that reveal the cooperative roles of hydrophobic, fluorophilic, and local polar interactions.

## 2 Results and Discussion

### 2.1 Design and Characterization of Polymers

We synthesized hypercrosslinked networks via the condensation of BCMBP and BTF using FeCl_3_ as a polymerization catalyst (synthetic scheme shown in Figure S1). The molar ratios of BCMBP to BTF were 1:1, 2:1, and 3:1, resulting in polymers FHCP‐1, FHCP‐2, and FHCP‐3, respectively. A non‐fluorinated control polymer (BP‐HCP) was also prepared by the self‐condensation of BCMBP under identical conditions. The resulting polymers were washed with deionized water and then subjected to Soxhlet extraction in methanol. The polymers appeared as orange/dark brown solids with yields above 70%. The synthesis was repeated at least three times to ensure reproducibility.

**FIGURE 2:**
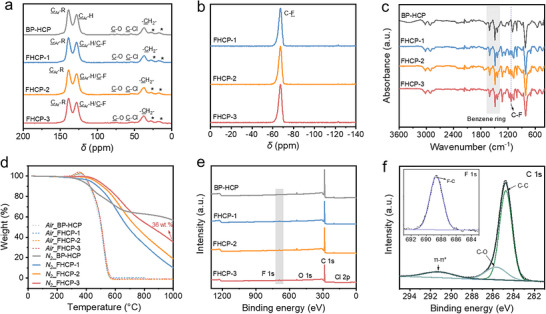
(a) ^13^C and (b) ^19^F solid‐state NMR spectra of FHCPs. * Indicate spinning sidebands. (c) FT‐IR spectra of all polymers. (d) TGA curves of all polymers in air and N_2_. (e) XPS survey spectra, and (f) high‐resolution C 1s spectrum of FHCP‐3, with the F 1s spectrum (inset) providing additional insight into fluorine incorporation.

^13^C cross‐polarization/magic‐angle spinning solid‐state nuclear magnetic resonance (^13^C ssNMR) was employed to verify the structural features of BP‐HCP and the three fluorinated hypercrosslinked polymers (FHCP‐1, FHCP‐2, and FHCP‐3) (Figure 2a). The peaks at 128 and 139 ppm correspond to aromatic carbon (C_Ar_‐H) and substituted aromatic carbon (C_Ar_‐R), respectively. Signals at 64 and 55 ppm correspond to C─O and C─Cl environments originating from trace hydrolysis and partially unreacted BCMBP side‐arms [31]. The broad peak around 37 ppm is attributed to the methyl bridges connecting the aromatic rings upon crosslink formation. Although the characteristic C─F resonance (∼125 ppm) overlaps with the strong aromatic carbon signals [17], fluorine incorporation into the FHCP networks is confirmed by ^19^F ssNMR (Figure 2b), which shows a distinct peak at −67 ppm corresponding to C─F groups [32]. Fourier‐transform infrared spectroscopy (FTIR) spectra (Figure 2c) further verified the desired network formation. The FHCPs gave distinct bands around 1132 cm^−1^, corresponding to C─F stretching vibrations [33], which were absent in the BP‐HCP control. Additionally, BP‐HCP exhibited a hydroxyl peak around 3200–3600 cm^−1^, which was not observed in the FHCP spectra, suggesting that fluorine incorporation effectively reduces the adsorption of moisture by enhancing network hydrophobicity. Thermogravimetric analysis (TGA) of BP‐HCP under N_2_ atmosphere showed that the onset of decomposition was ∼400°C, and almost complete degradation occurred at 1000°C (Figure 2d). The decomposition of FHCP‐1 exhibited a two‐stage process at temperatures of ∼300 C and ∼400°C, likely due to less dense crosslinking since the crosslinker (BCMBP) to BTF ratio was limited to a 1:1 ratio. The deterioration of FHCP‐2 and FHCP‐3 began at about 400°C, similarly to BP‐HCP. Among them, FHCP‐3 retained a substantial residual weight of 36 wt.% at 1000°C, demonstrating its high thermal stability. Network BP‐HCP had the largest residual weight, likely owing to higher crosslinking densities compared to FHCP networks incorporating BTF. When heated in an air atmosphere, all HCPs exhibited a weight increase at 290°C due to oxidation [34]. However, all networks started to decompose at 350°C and were completely thermally degraded at 575°C. The high temperatures at which degradation onset occurred in both N_2_ and air demonstrated high thermal stabilities for all networks.

X‐ray photoelectron spectroscopy (XPS) was performed to clarify the nature of chemical bonding in FHCPs. The survey spectrum showed four distinct peaks at 287, 688, 531, and 200 eV, corresponding to C 1s, F 1s, O 1s, and Cl 2p, respectively (Figure 2e). As shown in Figure 2f, the high‐resolution C 1s spectrum of FHCP‐3 can be deconvoluted into three peaks with binding energies of 284.7 and 285.7 eV, corresponding to C─C/C═C and C─O bonds, respectively, along with a broad and low‐intensity *π*–*π* shake‐up feature appearing around 291.2 eV [35]. In F HCPs, C─F was expected to give a peak at 286.0–286.5 eV but was not clearly distinguishable due to spectral overlap with C─O. The F 1s spectrum of FHCP‐3 shows one peak for organic F─C bonds at 688.5 eV. The four hypercrosslinked polymers' chemical features are validated by their C 1s and F 1s XPS spectra (Figure S2). The XPS elemental composition analysis showed that the surface fluorine contents of FHCP‐1, FHCP‐2, and FHCP‐3 were 1.49 wt.%, 1.75 wt.%, and 2.85 wt.%, respectively (Table S1). The strongly electron‐withdrawing –CF_3_ of BTF significantly reduces electrophilic reactivity, resulting in reduced incorporation into the network compared to theoretical values. Increased BCMBP crosslinker ratios in the polymer formulation when moving from FHCP‐1 to FHCP‐3 appeared to slightly improve the incorporation of BTF into the FHCPs, resulting in higher fluorine contents, although less BTF was present overall [36, 37].

**FIGURE 3:**
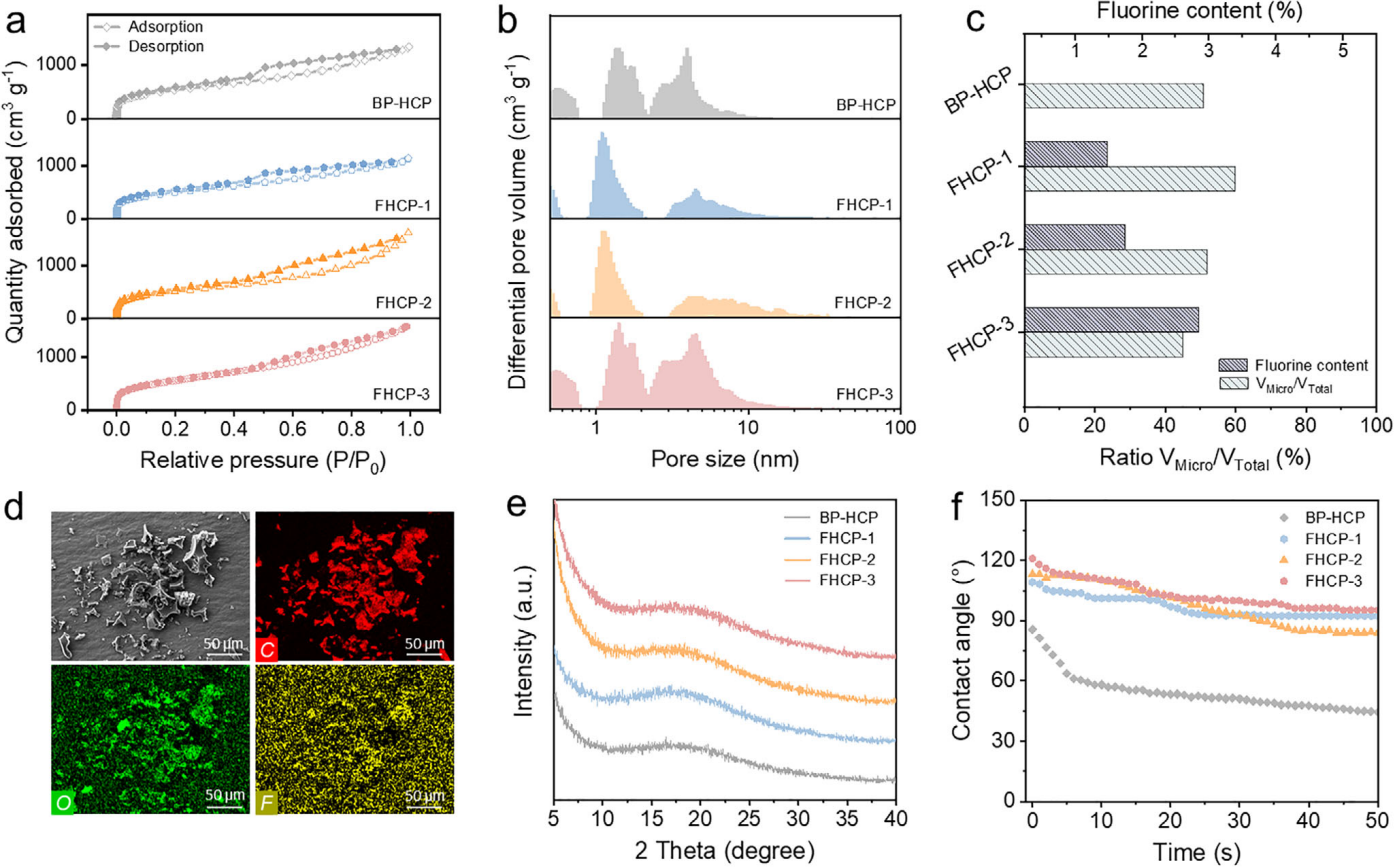
(a) N_2_ adsorption–desorption isotherms of all polymers measured at 77 K, (b) their QS‐DFT derived pore size distribution curves, and (c) fluorine contents and microporous volume ratios. (d) SEM image and elemental mapping (C, O, F) of FHCP‐3. (e) PXRD patterns of all polymers. (f) Water contact angle measurements over time.

Nitrogen adsorption/desorption measurements were conducted at 77 K to assess the porosity of the polymers. All four samples display H2‐type hysteresis loops, indicating a wide pore size distribution with relatively narrow pore necks (Figure 3a) [38]. The Brunner‐Emmett‐Teller specific surface areas (SSA_BET_) of BP‐HCP, FHCP‐1, FHCP‐2, and FHCP‐3 were calculated to be 1769 ± 79, 1591 ± 99, 1697 ± 51, and 1740 ± 61 m^2^ g^−1^, respectively, and were in good agreement with values obtained after self‐condensation of BCMBP reported previously [38, 39]. The incorporation of BTF did not appear to reduce the surface area significantly due to the relatively low BTF content.

The pore size distribution of all polymers was determined by applying quenched solid density functional theory (QS‐DFT) to the N_2_ adsorption branch. The most hierarchical and well‐developed porosity is found in FHCP‐3, which has a stronger mesopore contribution with a distinctive peak at ∼4.5 nm and a prominent micropore peak at ∼1.4 nm. On the other hand, BP‐HCP has a substantially decreased mesopore concentration and a strong micropore peak at around 1.3 nm. The micropore and mesopore contributions of FHCP‐1 and FHCP‐2 are between those of BP‐HCP and FHCP‐3, indicating intermediate porosity (Figure 3b). The micropore‐, mesopore‐, and total pore volume were also calculated via QS‐DFT and are summarized in Table S2. The total pore volumes of BP‐HCP, FHCP‐1, FHCP‐2, and FHCP‐3 were 3.08, 2.72, 2.64, and 3.01 cm^3^ g^−1^, respectively. Notably, the micropore volume of FHCP‐3 is 1.34 cm^3^ g^−1^, which is lower than in the other networks, suggesting that fluorination may allow further fine‐tuning of the micropore‐to‐meso‐/macropore ratio in FHCPs.

The ratio of micropore volume to total pore volume (V_Micro_/V_Total_) can be used to describe the degree of microporosity. The V_Micro_/V_Total_ values for the fluoropolymers FHCP‐1, FHCP‐2, and FHCP‐3 were 60%, 52%, and 45%, respectively, indicating an increasing fraction of mesopores (Figure 3c). Concurrently, the fluorine content increased from 1.49 wt.% to 2.85 wt.% (based on XPS data), suggesting that the introduction of ‐CF_3_ groups promoted mesopore formation. Mesopores are known to facilitate the uptake of adsorbates by enhancing guest molecule diffusion, while micropores are primarily responsible for the adsorption of small molecules [40, 41]. The tunable micropore‐to‐mesopore ratio in FHCPs plays a crucial role in adsorption, as a combination of micropores and mesopores is essential for effective adsorption. Micropores serve as strong binding sites, while mesopores enhance molecular accessibility and diffusion, thereby improving overall adsorption efficiency.

Scanning electron microscopy (SEM) coupled with energy‐dispersive X‐ray spectroscopy (EDS) was used to examine the morphology and elemental distribution of FHCP‐3 (Figure 3d), confirming uniform fluorine incorporation. The polymer presents an irregular particle morphology with coarse features. Powder X‐ray diffraction (PXRD) confirmed the amorphous nature of all polymers (Figure 3e), consistent with the highly crosslinked and non‐crystalline structure of the networks. Surface wettability was evaluated through time‐resolved contact angle measurements (Figure 3f). FHCPs exhibited significantly higher and more persistent contact angles than BP‐HCP, in line with their elevated fluorine content. The enhanced hydrophobicity helps reduce interfacial hydration and lowers the water‐layer resistance at the polymer surface, creating a more favorable environment for subsequent interactions between the fluorinated framework and PFOA. This interfacial modulation is consistent with reported C─F···F─C noncovalent motifs in fluorinated materials [42, 43].

### 2.2 Highly Efficient Removal of PFOA

**FIGURE 4:**
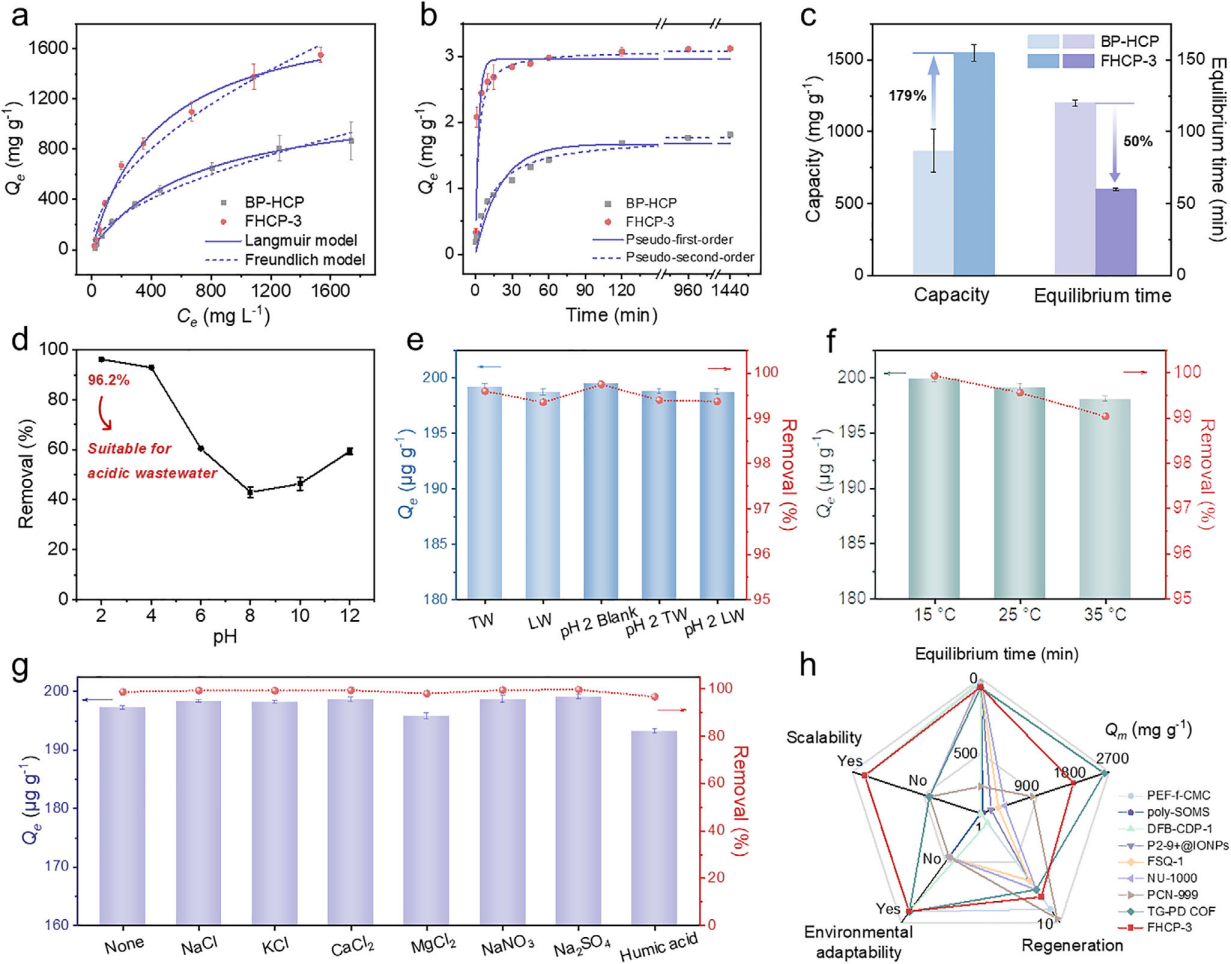
PFOA removal performances. (a) Equilibrium PFOA adsorption capacity, *Q*_e_, as a function of equilibrium PFOA concentration, *C*_e_, fitted with Langmuir and Freundlich models (initial concentration, *C*_0_: 25–2000 mg L^−1^; sorbent dosage: 0.3 g L^−1^; reaction time: 24 h; 25°C). (b) Sorption kinetics of PFOA with a *C*_0_ = 1 mg L^−1^, fitted with the pseudo‐first‐order model and pseudo‐second‐order model. (c) Comparison of adsorption capacity and equilibrium time of BP‐HCP and FHCP‐3. (d) Effects of solution pH on PFOA removal by FHCP‐3 in deionized water (*C*_0_ = 100 mg L^−1^; sorbent dosage: 0.3 g L^−1^). (e) Effects of water matrices on PFOA removal at low‐concentration aqueous conditions (*C*_0_ = 100 µg L^−1^; tap water (TW); lake water (LW); pH2 TW and pH2 LW represent acidified matrices). (f) Temperature‐dependent adsorption performance at 100 µg L^−1^ (15°C, 25°C, and 35°C). (g) Effects of background ions on PFOA removal by FHCP‐3 (TW was used as a matrix, containing 1 mm of anions or cations, 5 mg L^−1^ of humic acid (HA)). (h) Performances of FHCP‐3 compared with reported adsorbents from the literature [13, 15, 17, 26, 28, 46, 47, 48, 49, 50, 51, 52]. Unless otherwise stated, sorbent dosage was 0.5 g L^−1^, reaction time was 24 h, solution volume was 10 mL, and solution pH was ∼6.5. Error bars represent the standard deviation of three repeated experiments.

To evaluate adsorption capacity, BP‐HCP and FHCP samples were immersed in aqueous solutions of PFOA with varying initial concentrations and agitated for 24 h. PFOA was selected as a model PFAS due to its well‐characterized physicochemical properties and its widespread use in mechanistic adsorption studies, allowing direct comparison with prior literature. The chemical structure of PFOA has been estimated to have molecular dimensions of 13 Å × 6.5 Å (Figure S3) [44]. However, in aqueous solutions, hydrophobic interactions between fluorocarbon chains result in the formation of PFOA aggregates, particularly at elevated concentrations. This suggests that mesopores may play a crucial role in facilitating PFOA diffusion and adsorption by accommodating molecular clusters. After adsorption, residual PFOA concentrations were analyzed using liquid chromatography‐mass spectrometry (LC‐MS), with quantification based on a calibration curve (Figure S4). Equilibrium adsorption isotherms of PFOA on all networks were derived and fitted using the Langmuir and the Freundlich models (Figure 4a and Figure S5). The corresponding adsorption parameters are presented in Table S3. The Langmuir model exhibited higher coefficients of determination (R^2^) compared to the Freundlich model, indicating monolayer adsorption on a relatively homogeneous surface. Network FHCP‐3 displayed the highest maximum PFOA adsorption capacity (*Q*_e_) of 1550 ± 60 mg g^−1^ (3.7 mmol g^−1^) at an initial PFOA concentration of 2000 mg L^−1^, with a theoretical maximum adsorption capacity (*Q*_m_) of 1956 mg g^−1^ (4.7 mmol g^−1^), determined via the Langmuir model. The outstanding adsorption performance of FHCP‐3 is attributed to its higher fluorine content and polarized porous framework, which led to halogen bonding with the adsorbate. The increased hydrophobicity due to high fluorine content also improves hydrophobic adsorbent‐adsorbate interactions [45].

Considering the textural properties outlined above, both pore structure and surface chemistry play crucial roles in determining the adsorption performance of these polymers. Despite their similar SSA_BET_, variations in pore structure and chemical composition lead to distinct adsorption behaviors. Among the fluorinated polymers, FHCP‐3 exhibited the highest mesopore‐to‐total pore volume ratio (55%) according to QS‐DFT analysis, which is expected to facilitate enhanced PFOA diffusion and adsorption capacity. This observation supports the hypothesis that pore size plays a critical role in the adsorption process, where larger mesopores reduce diffusion resistance, leading to improved adsorption performance. In contrast, FHCP‐1 (40%) and FHCP‐2 (48%) exhibited lower mesopore‐to‐total pore volume ratios, indicating a more confined pore structure. This reduced mesopore volume, coupled with a narrower mesopore distribution, may hinder PFOA diffusion and potentially limit adsorption efficiency relative to that in FHCP‐3. The notably lower adsorption performance of BP‐HCP further supports this hypothesis, as its predominantly microporous nature restricts molecular access and diffusion. Additionally, while FHCPs and BP‐HCP have similar SSA_BET_ and micropore‐to‐total pore ratios, FHCP‐3 achieves significantly higher PFOA adsorption.

The exceptional performance of FHCP‐3 motivated further investigation into the kinetics of the adsorption process. In general, the lower the initial concentration of PFOA, the more challenging the adsorption process becomes [46]. As shown in Figure 4b, FHCP‐3 exhibited rapid adsorption kinetics at 1 mg L^−1^ (1 ppm) PFOA, reaching equilibrium within 60 min, whereas BP‐HCP required at least 120 min to approach equilibrium. Based on the pseudo‐second‐order kinetic model, the adsorption rate constant (k_2_) of FHCP‐3 was calculated to be 1.60 × 10^−3^ g mg^−1^ min^−1^, with an improved R^2^ = 0.98 compared to the pseudo‐first‐order kinetic model (R^2^ = 0.97, Figures S6 and S7). The better agreement with the pseudo‐second‐order model, together with the short equilibration time, suggests that the overall rate is dominated by surface‐controlled interactions rather than by a single diffusion‐limited step. Given the hierarchical micro‐mesoporous structure of FHCP‐3, film diffusion and intraparticle transport are expected to be fast, and thus diffusion contributes to the overall kinetics but does not act as the sole rate‐determining process. The fluorine‐decorated network exhibited markedly enhanced performance in the removal of PFOA, achieving *Q_e_* approximately 179% higher than that of BP‐HCP and reaching adsorption equilibrium in half the time (Figure 4c). It is important to note that factors such as initial adsorption concentration, adsorbent dosage, and solution volume significantly influence adsorption kinetics. The adsorption capacity (*Q*_m_) of samples follows an increasing trend from BP‐HCP to FHCP‐3, highlighting how mesopore structure and fluorine incorporation enhance adsorption performance. Due to its exceptional adsorption capacity, FHCP‐3 is among the best‐performing adsorbents for PFOA capture, outperforming numerous COFs [15, 28], MOFs [13, 46, 47], and other adsorbents such as SOMS [48], DFB‐CDP [49, 50], GenX [26], and PEF‐f‐CMC [51] (Table S4).

The PFOA removal performance of FHCP‐3 was evaluated over a wide pH range (2–12). FHCP‐3 achieved exceptionally high removal efficiency (96.2%) at an initial concentration of 100 mg L^−1^ under strongly acidic conditions (Figure 4d), confirming its effectiveness in highly contaminated wastewater. Zeta‐potential analysis (Figure S8) revealed that FHCP‐3 is positively charged at pH 2–4, potentially due to the trace oxygenated species and residual chloromethyl groups seen in XPS, which promotes uptake of anionic PFOA, whereas surface deprotonation at alkaline pH leads to reduced removal efficiency [53, 54]. The sustained adsorption even under strongly negative potentials indicates that fluorophilic and hydrophobic interactions dominate the uptake process, with electrostatic effects contributing only to the pH‐dependent variation.

The present study is designed to establish intrinsic sorbent performance and mechanistic behavior under controlled laboratory conditions; accordingly, low‐concentration experiments were conducted within the validated analytical range of the LC‐MS method to enable reliable comparison across different aqueous matrices. Tests initially performed at 10 µg L^−^^1^ (ppb) fell below the limit of quantification (LOQ), and subsequent evaluations were therefore conducted at 100 µg L^−^^1^ to ensure reliable quantification. Residual PFOA concentrations were quantified by LC‐MS using an external calibration curve, and representative raw chromatograms of the standards are provided in the (Figures S9–S12) to demonstrate retention‐time stability and linear response. As shown in Figure 4e, FHCP‐3 maintained excellent removal efficiency across different water matrices. In neutral tap water (TW) and lake water (LW), removal efficiencies exceeded 99.5% and 99.3%, respectively. Upon acidification to pH 2, FHCP‐3 consistently achieved >99.3% removal in both TW and LW, with only minimal matrix effects, again reflecting the dominance of fluorophilic and hydrophobic interactions in driving PFOA uptake. Across the investigated temperature range, the adsorption capacity showed only limited variation (Figure 4f). This observation is further supported by the temperature‐dependent adsorption isotherms measured at 30°C, 40°C, and 50°C (Figure S13), which show a general decrease in equilibrium uptake with increasing temperature. To further quantify the temperature effect, temperature‐dependent adsorption kinetics were measured at 30°C, 40°C, and 50°C (Figure S14a–c). In all cases, rapid uptake was followed by a gradual approach to equilibrium, and the pseudo‐second‐order model provided a better fit than the pseudo‐first‐order model (Table S5). While the equilibrium uptake remained comparable across the investigated temperatures, the pseudo‐second‐order rate constant and initial adsorption rate increased markedly with temperature. Arrhenius analysis gave an apparent activation energy of 53.7 kJ mol^−1^ (Figure S14d), indicating that the uptake process is thermally activated. These results show that, although the equilibrium uptake is only weakly temperature‐dependent, the adsorption kinetics are significantly accelerated at elevated temperatures.

FHCP‐3 also exhibited strong tolerance to coexisting ionic and organic species (Figure 4g). Monovalent and divalent cations (Na^+^, K^+^, Ca^2+^, and Mg^2+^) and common anions (Cl^−^, NO_3_^−^, and SO_4_^2−^) produced negligible interference, with removal efficiencies above >97% at 100 µg L^−1^ PFOA. Given an initial concentration of 100 µg L^−1^, a removal efficiency of 99% corresponds to a near‐LOQ residual concentration of approximately 0.4 µg L^−1^, which reflects the mathematical relationship between removal efficiency and starting concentration rather than a limitation of adsorption affinity. Humic acid (HA, 5 mg L^−1^) caused a modest reduction in PFOA uptake (96.5%), attributable to some competitive hydrophobic association and partial pore obstruction. The influence of the overall ionic strength was examined by varying NaCl concentration from 0 to 100 mmol L^−1^ (mm). FHCP‐3 preserved removal efficiencies above 99.1% across the entire salinity range (Figure S15). A modest enhancement at intermediate salinity (1–10 mm) is consistent with moderate electric‐double‐layer compression facilitating PFOA approach, whereas high salinity (100 mm) produced no significant performance loss. From the low‐concentration experiments, distribution coefficients (*K*_D_) and its log‐transformed value (log*K*_D_) were evaluated to quantify the sorption affinity of FHCP‐3 (Table S6). Because *K*_D_ and log*K*_D_ normalize both sorbent dosage and equilibrium concentration, they provide a reliable measure of intrinsic sorption affinity [55, 56]. FHCP‐3 exhibited consistently high sorption affinity across all tested matrices, with *K*_D_ = 600 L g^−1^ (log*K*_D_ = 2.78) in deionized (DI) water and slightly lower log*K*_D_ values in tap and lake water (2.49–2.69), indicating minimal interference from background ions. Under acidic conditions, log*K*_D_ increased to 2.89 in DI water and remained high in natural waters (2.50–2.52). These findings underscore the strong potential of FHCP‐3 for treating PFOA‐contaminated waters, highlighting the effectiveness of its fluorinated framework in sustaining high affinity in complex conditions.

### 2.3 Adsorbent Recovery and Regeneration

The PFOA‐loaded FHCP‐3 (PFOA@FHCP‐3) was characterized after adsorption (PFOA *C*_0_: 2000 mg L^−1^, adsorption time: 24 h, temperature: 25°C) using FTIR, XPS, and N_2_ sorption analysis. A PFOA concentration of 2000 mg L^−1^ was selected to ensure that FHCP‐3 was evaluated under sorbent‐saturating conditions. The FTIR spectra indicate that the basic structure of the polymer remains unchanged after adsorption, with the appearance of new peaks associated with PFOA at 1779 and 1686 cm^−1^ (C═O), and 1242 and 1207 cm^−1^ (‐CF_2_) (Figure S16), confirming adsorption and the structural integrity of FHCP‐3. Additionally, XPS analysis (Figure S17) revealed a 5.5‐fold increase in fluorine content after adsorption, from 2.0 at.% to 13.2 at.%, highlighting adsorption efficiency. N_2_ adsorption–desorption isotherms and pore size distribution curves of FHCP‐3 before and after PFOA adsorption (Figure S18) reveal critical insights into the adsorption process and material stability. The SSA_BET_ decreased by ∼26% to 1331 m^2^/g, indicating partial pore blockage. The mesopore volume dropped from 1.67 to 1.16 cm^3^ g^−1^, while micropore volume declined from 1.34 to 0.88 cm^3^ g^−1^. The greater reduction in mesopore volume suggests their role as diffusion pathways and possible sites for PFOA aggregation, while micropores serve as high‐affinity adsorption sites. Notably, the fluorine‐rich pore environment of FHCP‐3 enhances adsorption through fluorophilic interactions, stabilizing PFOA molecules within the porous framework. Minimal shifts in the pore size distribution confirm that the framework remains intact, while the uniform decline in pore volume across all sizes suggests that PFOA is accommodated in both micro‐ and mesopores. This synergy between hierarchical porosity and fluorine‐mediated interactions underpins FHCP‐3′s superior adsorption performance, highlighting its potential for sustainable water treatment processes targeting perfluorinated compounds.

Adsorbent regenerability is a crucial characteristic for sustainable and cost‐effective water treatment. To assess the regenerability of our adsorbent, FHCP‐3 was washed with methanol before reuse in subsequent PFOA adsorption cycles. Figure S19 illustrates the PFOA removal efficiency of FHCP‐3 over six consecutive adsorption‐regeneration cycles upon exposure to a 100 mg L^−1^ PFOA solution. The removal efficiency varied from 97% to 86% over the six regeneration cycles, with *Q*_e_ ranging from 200 to 113 mg g^−1^. Notably, even after six cycles, the adsorption capacity remained high, indicating the promising recyclability of the adsorbent and further highlighting the excellent stability of FHCP‐3. The FTIR spectra compare FHCP‐3 after six adsorption cycles with the regenerated sample following methanol washing, confirming the removal of adsorbed PFOA (Figure S20). The N_2_ adsorption–desorption isotherms yielded a slight decrease in the BET surface area from 1689 to 1613 m^2^ g^−1^ after regeneration, while the overall porosity remains largely preserved (Figure S21), demonstrating the structural stability of the polymer network during repeated use. A schematic representation of the PFOA adsorption and desorption process on BP‐HCP and FHCP‐3 is provided in Figure S22 to illustrate the fluorine‐enabled interfacial interactions involved in the regeneration process. Although methanol is convenient and effective for laboratory‐scale regeneration, its suitability for larger‐scale operation would depend on solvent recovery, process safety and cost considerations. Future work may therefore explore alternative alcohol‐based or mixed‐solvent systems that could offer more practical and sustainable regeneration conditions.

To further assess the potential for practical deployment, we compared the costs of FHCP‐3 and various commercial and research‐grade sorbents used for PFAS remediation. As summarized in Table S7, the estimated materials cost of FHCP‐3 is substantially lower than that of fluorinated adsorbents and remains competitive with specialized activated carbons. It should be noted that the calculated material costs are for comparative purposes only and consider lab‐scale production. Beyond cost considerations, fluorinated COFs and MOFs generally rely on multistep linker synthesis or solvothermal crystallization, leading to processing burdens and hindering scale‐up. FHCP‐3 is obtained in a one‐pot Friedel‐Crafts polymerization of inexpensive commodity monomers. To demonstrate the practicality of this synthetic route, repeated scaled‐up syntheses were performed using large reaction vessels, enabling the accumulation of approximately 100 g of FHCP‐3 under standard laboratory conditions (Figure S23). Structurally, FHCP‐3 offers a high density of accessible fluorine functionalities dispersed throughout a hierarchical micro‐mesoporous framework, enabling rapid mass transport and strong fluorophilic interactions that complement its synthetic and economic advantages. Taken together, FHCP‐3 demonstrates high adsorption capacity, rapid equilibration, robust regenerability, environmental adaptability, and favorable cost when compared with reported adsorbents (Figure 4h).

### 2.4 Mechanistic Insights

**FIGURE 5:**
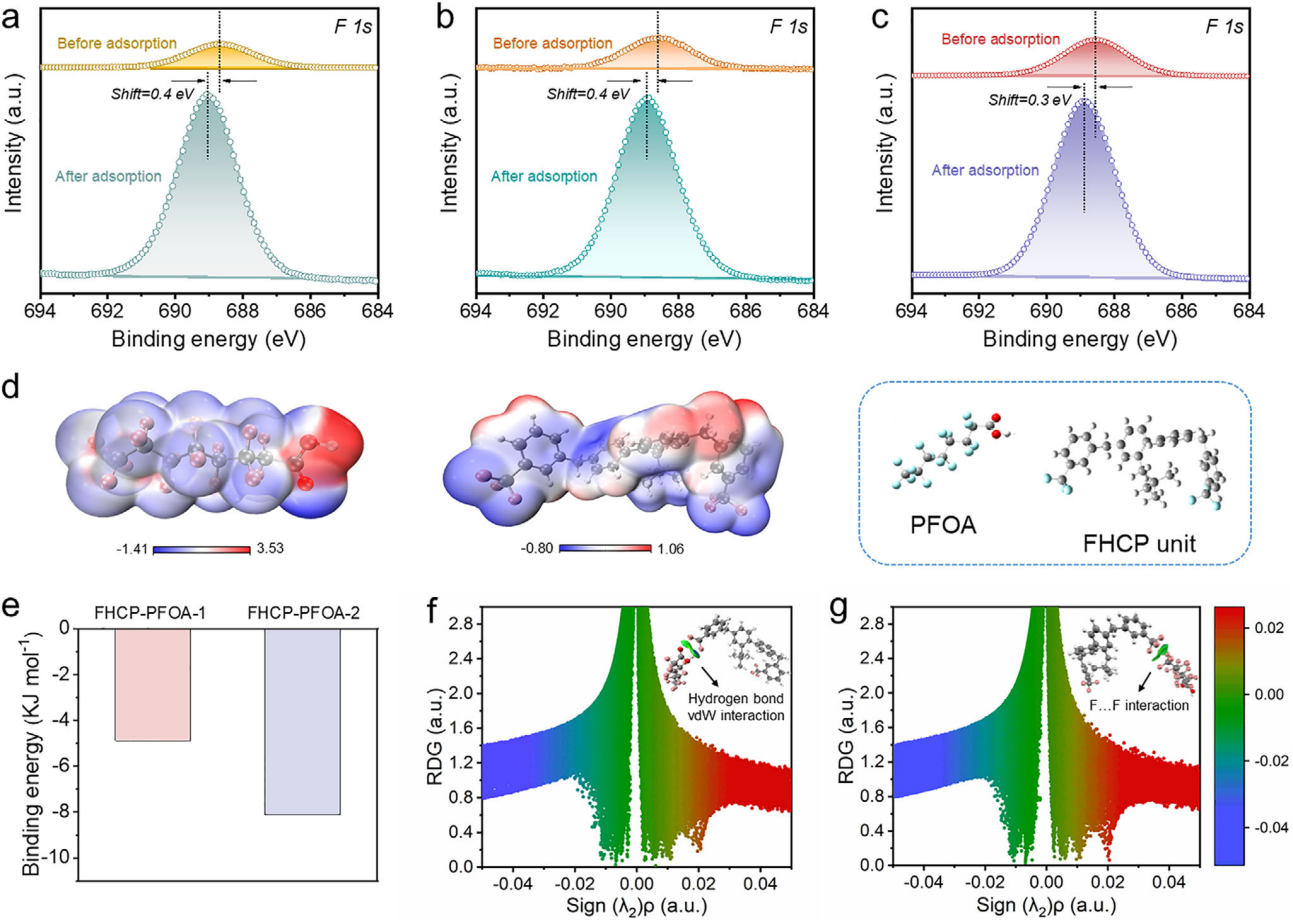
(a–c) F 1s XPS spectra of FHCP‐1, FHCP‐2, and FHCP‐3 before and after PFOA adsorption. (d) ESP maps of a PFOA molecule and an FHCP repeating unit. (e) Calculated binding energies for two adsorption configurations between FHCP and PFOA, shown in Figure S27. (f,g) RDG analyses for the two complexes.

To investigate the molecular basis of PFOA recognition by our FHCPs, we combined surface spectroscopic characterization with theoretical modeling. F 1s XPS spectra of FHCP‐1, FHCP‐2, and FHCP‐3 exhibited strong C‐F signals at 688.68, 688.58, and 688.58 eV, respectively [42] Upon exposure to PFOA, all samples showed consistent binding energy shifts of 0.3–0.4 eV (Figure 5a–c), which reflects changes in the local electronic environment of −CF_2_/−CF_3_ groups rather than the magnitude of a specific interaction such as hydrogen bonding or fluorophilic contacts [56]. Complementary C 1s spectra revealed changes in the intensity and position of the ‐CF_3_ peaks after adsorption (Figure S24), reflecting alterations in the local chemical environment. In parallel, FTIR spectra exhibited characteristic stretching vibrations of ‐CF‐, ‐CF_2_‐, and ‐CF_3_ groups in the 1100–1300 cm^−1^ region [33]. A red‐shift of 12 cm^−1^ in the C‐F band upon PFOA binding (Figure S25) further supports the existence of fluorine–fluorine interactions between the polymer and the adsorbate.

To elucidate the molecular origin of the observed affinity between FHCP and PFOA, we performed a series of DFT calculations, including electrostatic potential (ESP) mapping, structural optimization, binding energy estimation, and noncovalent interaction analysis. The ESP surface of PFOA reveals a pronounced electron‐deficient region at the carboxylic headgroup, while the FHCP framework exhibits polarized electron‐rich domains distributed along the fluorinated polymer backbone (Figure 5d). These complementary charge distributions suggest a favorable alignment for directional polar interactions [43]. Further ESP mapping of the two optimized complexes (Figure S26) illustrates distinct modes of interfacial charge redistribution. In both configurations, electron‐deficient zones on PFOA are positioned adjacent to electron‐rich regions on FHCP, reinforcing the concept of site‐specific association via charge complementarity [57]. Rather than classical electrostatic attraction between formal charges, the interaction is mediated by dipole–dipole alignment and local polarization effects arising from the fluorine‐decorated backbone. These results indicate that the spatial arrangement of ESP plays a key role in guiding selective binding between FHCP and PFOA.

Structural optimization of the FHCP‐PFOA complexes revealed two distinct and thermodynamically favorable binding configurations, each stabilized by different noncovalent forces. In FHCP‐PFOA‐1, a strong hydrogen bond forms between the carboxylic acid moiety of PFOA and a hydrogen‐donating site on the FHCP backbone, with an intermolecular distance of 1.88 Å and a binding energy (*∆E*) of −8.12 KJ mol^−1^. In contrast, FHCP‐PFOA‐2 features a fluorine–fluorine contact between adjacent CF_3_ groups at a distance of 2.85 Å and a *∆E* of −4.91 KJ mol^−1^ (Figure 5e and Figure S27). Both modes exhibit negative binding energies and negative Gibbs free energies (Δ*G*), confirming that adsorption proceeds spontaneously under ambient conditions [42]. Notably, although hydrogen bonding contributes more significantly to the binding enthalpy, the smaller *∆G* difference of 0.76 kcal mol^−1^ between the two modes suggests that fluorophilic contacts, while individually weaker, remain energetically relevant and operate cooperatively with hydrogen bonding and hydrophobic interactions.

**FIGURE 6:**
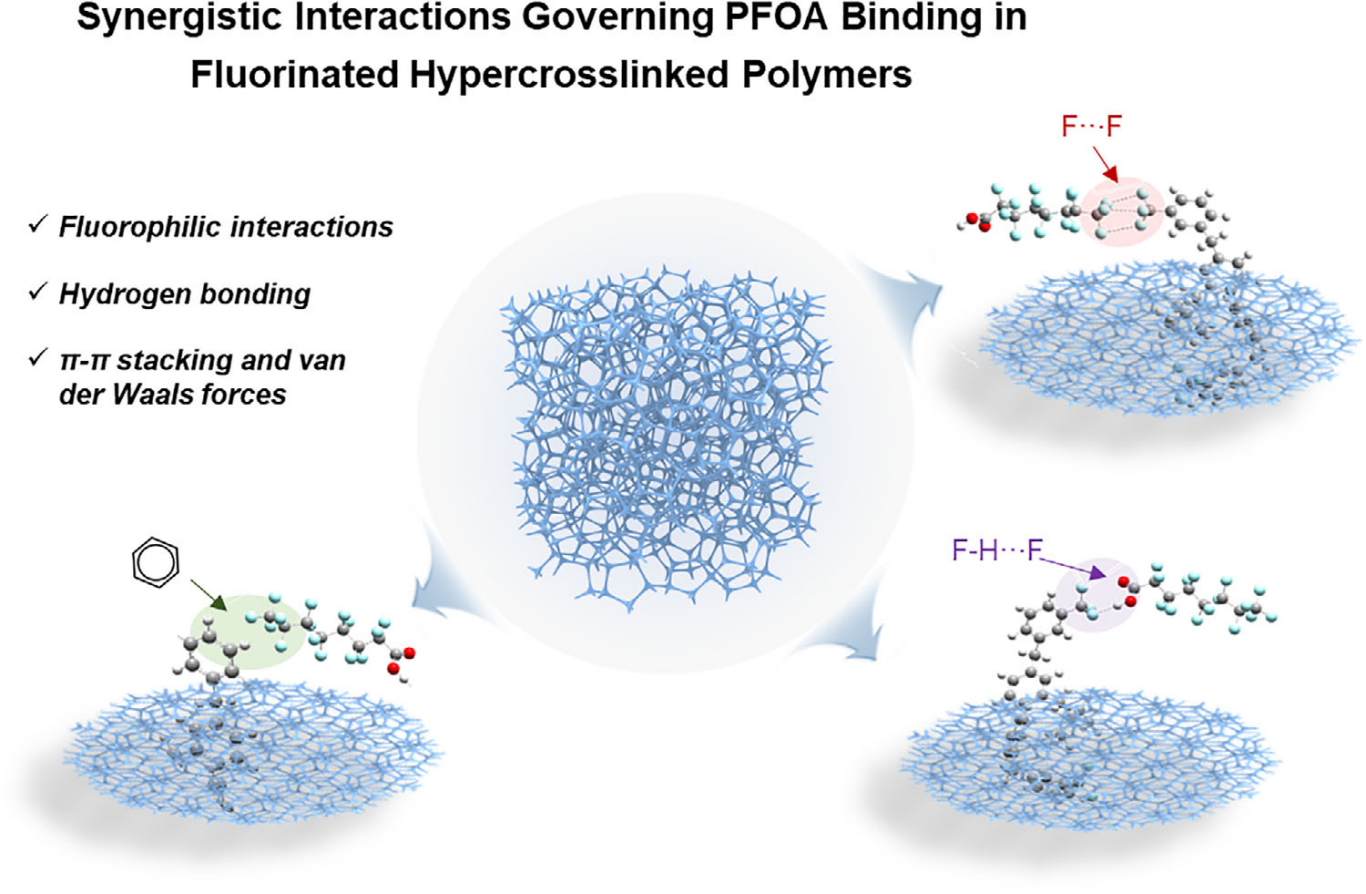
Schematic illustration of the synergistic noncovalent interactions governing PFOA binding within the fluorinated hypercrosslinked polymer network.

Visual evidence for the multivalent binding mechanism was obtained through reduced density gradient (RDG) analysis, which reveals the spatial distribution of noncovalent interactions in the optimized complexes. In FHCP‐PFOA‐1 (Figure 5f), pronounced green isosurfaces appear between the ─COOH group of PFOA and nearby hydrogen‐donating sites on the polymer, consistent with strong and localized hydrogen bonding. In contrast, FHCP‐PFOA‐2 (Figure 5g) displays more diffuse green regions between adjacent CF_3_ units, indicative of structurally persistent fluorine–fluorine contacts. These fluorophilic contacts, made possible by the densely fluorinated and crosslinked polymer environment, provide spatial complementarity that guides the selective binding of perfluorinated contaminants. Taken together, the RDG visualizations support a cooperative binding model wherein directional hydrogen bonding is complemented by fluorophilic interactions and hydrophobic confinement, collectively promoting efficient PFOA sequestration within the fluorinated porous polymer matrix. A schematic illustrating these cooperative noncovalent interactions is shown in Figure 6, summarizing the mechanisms of adsorption revealed by both experiments and simulations and reinforcing the central role of these interactions in governing PFOA capture. Rather than relying on a single interaction type, the FHCP frameworks enable cooperative interactions, distinguishing FHCP‐3 from many previously reported fluorinated adsorbents that primarily depend on surface fluorination or limited fluorophilic contacts.

## 3 Conclusion

Removing PFOA from contaminated water is a challenging task. State‐of‐the‐art adsorbents often lack sufficient PFOA removal capacity and exhibit limited regenerability. In this study, we demonstrated through systematic PFOA adsorption tests that the fluorinated hypercrosslinked polymer FHCP‐3 is an effective adsorbent for removing PFAS from aqueous media. FHCP‐3 exhibits an impressive adsorption capacity for PFOA, reaching up to 1550 mg g^−1^ (3.7 mmol g^−1^), with rapid kinetics achieving equilibrium within 1 h in a solution of 1 mg L^−1^ PFOA. Moreover, FHCP‐3 demonstrated outstanding regenerability and reusability, a first in the PFAS@FHCPs field, maintaining nearly undiminished removal and recovery rates after six consecutive adsorption–desorption cycles. Under low‐concentration aqueous conditions (100 µg L^−1^), FHCP‐3 consistently achieved >99% PFOA removal across varied water matrices, ionic strengths, temperatures, and background species, demonstrating excellent robustness. The superior performance of FHCP‐3 relative to BP‐HCP originates from fluorine incorporation, which enhances adsorption through cooperative fluorophilic and hydrophobic interactions, as further supported by DFT and ESP analyses. Although this work focuses on PFOA, the identified mechanisms may be broadly applicable to other PFAS molecules. Extending this strategy to PFOS and short‐chain PFAS will form an important part of future studies. Overall, FHCP‐3 represents a robust and hydrolytically stable platform for water remediation, providing a strong foundation for the development of next‐generation adsorbents with enhanced capability to address persistent organic pollutants under trace‐level regulatory scenarios.

## 4 Experimental Section

### 4.1 Materials

All chemicals and reagents are commercially available and used without further purification. 1,2‐Dichloroethane (DCE, ≥99%), 4,4′‐bis(chloromethyl)‐1,1′‐biphenyl (BCMBP, 95%), and iron (III) chloride (FeCl_3_, 97%) were purchased from Sigma–Aldrich. α,α,α‐Trifluorotoluene (BTF, >98%) was purchased from Tokyo Chemical Industry Co., Ltd. Methanol (≥99.8%) was purchased from Fisher Scientific. Perfluorooctanoic acid (PFOA, 96%) was purchased from Shanghai Merrell Chemical Technology Co., Ltd.

### 4.2 Synthesis of Hypercrosslinked Polymer (BP‐HCP)

BCMBP (1 mmol, 0.251 g) was dissolved in DCE (10 mL) at room temperature over stirring. Once fully dissolved, FeCl_3_ (1.5 mmol, 0.243 g) was introduced, leading to the rapid formation of a solid. After 15 min, a reflux condenser was connected, and the reaction mixture was heated to 80°C for 24 h. Upon cooling, the brown solid was collected and rinsed with 50 mL of methanol using a Buchner funnel. It was then further Soxhlet extracted with methanol overnight. The polymer was left to air dry in the fume cupboard at room temperature overnight to remove excess methanol, followed by drying in a vacuum oven at 80°C overnight. The resulting BP‐HCP was lightly ground with a pestle and mortar and used for subsequent analyses.

### 4.3 Synthesis of Fluorinated Hypercrosslinked Polymers (FHCPs)

We produced three polymers by varying the molar ratios of BCMBP:BTF (1:1, 2:1, and 3:1). Taking the 1:1 ratio as an example, BCMBP (1 mmol, 0.251 g) and BTF (1 mmol, 0.146 g) were dissolved in DCE (10 mL) at room temperature over stirring. Upon complete dissolution, FeCl_3_ (1.5 mmol, 0.243 g) was added, and a solid was formed almost immediately. After 15 min, a reflux condenser was attached to the reaction and the solution was heated to 80°C for 24 h. After cooling, the resulting solid was washed with 50 mL of methanol in a Buchner funnel before being washed overnight via Soxhlet extraction, again with methanol. The polymer was air‐dried in a fume cupboard at room temperature overnight to remove excess methanol, followed by drying in a vacuum oven at 80°C overnight. The dry FHCP was lightly ground with a pestle and mortar. The final polymers were named FHCP‐1, FHCP‐2, and FHCP‐3, referring to the BCMBP:BTF ratios of 1:1, 2:1, and 3:1, respectively.

### 4.4 Characterization

FTIR spectra were collected using a Tensor II FTIR spectrometer (Bruker) equipped with a Bruker Optics Platinum ATR module. The spectra were recorded and analyzed using the integrated OPUS 7.5 software. The ^13^C ssNMR and ^19^F ssNMR experiments were measured with a Bruker Avance NEO 500 wide‐bore system (Bruker BioSpin) using a 4 mm triple resonance magic angle spinning probe. TGA was performed using a Discovery TGA (TA Instruments). Approximately 6 mg of samples were heated under dry N_2_ or air gas flow (25 mL min^−1^) at a ramp rate of 10°C min^−1^ from room temperature to 1000°C. XPS was performed on a Nexsa Photoelectron Spectrometer (Thermo Scientific). All measurements were performed using Al‐Kα X‐rays with a spot size of 400 µm. The SSA_BET_ was determined using the BET equation applied to the N_2_ adsorption–desorption isotherms recorded with a Micromeritics 3Flex instrument at 77 K. The samples were dried at 120°C for 12 h before analysis.

### 4.5 Quantification of PFOA

For the uptake assay, PFOA concentrations in the test solutions before and after adsorption were determined by LC‐MS. For each batch experiment, 2 mL of supernatant was collected and filtered using 0.22 µm polyethersulfone (PES) syringe filters. To minimize the effect of filter sorption, the initial 1 mL was discarded and the remaining 1 mL was collected for analysis. LC‐MS analyses were performed on a Vanquish Core high‐performance liquid chromatography system coupled to the ESI source of an Orbitrap Exploris 120 mass spectrometer using an Acclaim 120 C18, 2.1 × 150 mm, 3 µm column (all from Thermo Fisher Scientific). Water and acetonitrile/water 95:5, both modified with 0.1% formic acid, served as mobile phase A and B, respectively. 5 µL of the appropriately diluted liquid samples were injected and the components separated with a linear gradient from 60% to 70% B in 2 min, followed by an isocratic column cleaning (2 min at 100% B) and re‐equilibration step (4 min at 60% B). The flow rate was 0.5 mL min^−1^ and the column oven temperature was set to 40°C. Based on calibration curves and signal‐to‐noise ratios, the LOQ for PFOA under the applied LC–MS conditions was determined to be 0.5  µg L^−1^. Concentrations at or below this value approach the lower boundary of reliable quantification and are therefore reported as near‐LOQ values to avoid overinterpretation. Within the validated quantification window, the analytical uncertainty was approximately 5% at low concentrations and around 15% at the upper boundary of reliable quantification concentrations (>800 mg L^−1^). High‐resolution ESI mass spectra were recorded in negative ion mode in the range of *m/z* 100–1000 at a resolution of 60 000. Quantification was achieved via linear calibration functions calculated with at least 6 calibration points. The analytical error was 5% at low concentrations and estimated to be around 15% at high concentrations (>800 mg L^−1^).

## Conflicts of Interest

The authors declare no conflicts of interest.

## Supporting information




**Supporting File**: smll74048‐sup‐0001‐SuppMat.docx.

## Data Availability

The data that support the findings of this study are available from the corresponding author upon reasonable request.
