# Supplementary material for: Fluorinated Hypercrosslinked Polymers with Exceptional Capacity for Perfluorooctanoic Acid Removal from Water
Source: Small. 2026 Jun 4;22(43):e09398. doi: 10.1002/smll.202509398 (PMC13432669; doi:10.1002/smll.202509398)
Supplement: Supplementary file 1 — Supporting File: smll74048‐sup‐0001‐SuppMat.docx. [file SMLL-22-e09398-s001.docx]

Supporting Information

**Fluorinated Hypercrosslinked Polymers with Exceptional Capacity for Perfluorooctanoic Acid** **Removal from Water**

*Mingqing Yu,^1,2^ Anna Fabisikova,^3^* *Paul Schweng,^2^ Martin Zehl,^3^ Huajie Yu,^1^ Yaozu Liao,^1,^ * Robert T. Woodward ^2,^**

^1^ State Key Laboratory of Advanced Fiber Materials, College of Materials Science and Engineering, Donghua University, Shanghai 201620, China

^2^ Institute of Materials Chemistry and Research, Faculty of Chemistry, University of Vienna, Währinger Straße 42, Vienna 1090, Austria

^3^ Department of Analytical Chemistry, Faculty of Chemistry, University of Vienna, Währinger Straße 38, 1090 Vienna, Austria

*Corresponding authors’ email: yzliao@dhu.edu.cn; robert.woodward@univie.ac.at

**Table of Contents**

[1. General methods and instruments 1](#_Toc224604076)

[1.1 Sorption experiments 1](#_Toc224604077)

[1.2. Theoretical calculation 5](#_Toc224604078)

[2. Figures (S1-S27) 7](#_Toc224604079)

[2.1 Synthetic routes to FHCPs 7](#_Toc224604080)

[2.2. Structural characterisation of HCPs and FHCPs 7](#_Toc224604081)

[2.3. Adsorption performance 8](#_Toc224604082)

[2.4. Adsorbent recovery and regeneration 18](#_Toc224604083)

[2.5. Mechanistic insight 24](#_Toc224604084)

[3. Tables (S1-S7) 27](#_Toc224604085)

[4. Supplementary References 32](#_Toc224604086)

# 1. General methods and instruments

## 1.1 Sorption experiments

Prior to perfluorooctanoic acid (PFOA) adsorption experiments, the as-synthesised HCP and FHCP samples were washed with methanol several times, followed by drying in a vacuum oven at 80 ℃ overnight. PFOA adsorption experiments were performed in aqueous solutions with a mass-to-volume ratio, m/V, of 3/10 (3 mg of HCP in 10 mL of solution). After shaking for a certain time at room temperature, the supernatant was decanted, and the PFOA-loaded HCP samples (PFOA@FHCP-3) were washed with water and dried.

**Adsorption isotherms** were collected by exposing 3 mg of adsorbent to 10 mL of aqueous PFOA solution with initial concentrations, *C*_0_, of 25, 50, 100, 200, 400, 600, 1000, 1500, and 2000 mg L^−1^ in 25 mL vials. The solutions were mixed in a shaker at 300 rpm at room temperature. After 24 h, the (F)HCPs were removed via a syringe filter (0.22 μm, polyethersulfone (PES)) and diluted using deionised (DI) water for analysis. All solutions were agitated with a vortex mixer before analysis by liquid chromatography-mass spectrometry (LC-MS) to determine the equilibrium concentration, *C*_e_, after adsorption. Prior to sample analysis, fresh standard solutions were prepared for each calibration, and the calibration process was repeated until the correlation coefficient (R^2^) consistently reached 0.999 or higher. No other pretreatment was performed. All experiments were performed at least in triplicate and each measurement was collected twice. Average values are reported. Langmuir and Freundlich models were applied to fit the experimental data:

*Langmuir model: $\text{Q}_{\text{e}}\text{=}\frac{\text{(}\text{Q}_{\text{m}}\text{×}\text{k}_{\text{L}}\text{×}\text{C}_{\text{e}}\text{)}}{\text{1+}\text{k}_{\text{L}}\text{+}\text{C}_{\text{e}}}\text{ }\text{ }\text{ S1}$

Where *Q*_e_ (mg g^−1^) is the amount of PFOA absorbed at equilibrium, *Q*_m_ (mg g^−1^) is the maximum adsorption capacity of the adsorbent at equilibrium. *C_e_* (mg L^−1^) is the residual concentration of analytes at equilibrium, and *k*_L_ (L mg^−1^) is the Langmuir equilibrium constant.

*Freundlich model: $Q_{e}=C_{e}^{1/n}\times k_{F}$ S2

Where *Q_e_* (mg g^−1^) is the amount of PFOA adsorbed at equilibrium, *C_e_* (mg L^−1^) is the residual concentration at equilibrium. *k_F_* (mg g^−1^)(L mg^−1^)^1/n^ is the Freundlich constant. *n* is an indicator of the intensity of the adsorption.

**Adsorption kinetics** of (F)HCPs were evaluated upon the addition of 3 mg of polymer to a 1 mg L^−1^ aqueous PFOA solution. The solutions were then sampled at 1, 5, 10, 15, 30, 45, 60, 120, 960, and 1440 min (24 h). A control experiment was performed without the addition of adsorbents to evaluate any loss of PFOA from adsorption on to vial walls and/or filters, again for 24 h. All samples were syringe filtered (0.22 μm, PES) and diluted using DI water for analysis. All solutions were agitated with a vortex mixer before analysis by LC-MS to determine the equilibrium concentration, *C*_e_, after adsorption. No other pretreatment was performed. All experiments were performed at least in triplicate and each sample was measured twice. Average values are reported. The experimental data were fitted with pseudo-first-order and pseudo-second-order models.

*pseudo-first-order: $\text{Q}_{\text{t}}\text{=}\text{Q}_{\text{e}}\text{(}\text{1-}\text{e}^{\text{(-}\text{k}_{\text{1}}\text{×t)}}$) S3

Where *Q*_t_ (mg g^−1^) is the amount of analyte adsorbed at the given time t (min), *Q*_e_ (mg g^−1^) is the amount of PFOA adsorbed on the adsorbent at equilibrium, *k*_1_ (min^−1^) is the rate constant of adsorption.

*pseudo-second-order: $\text{Q}_{\text{t}}\text{=}\frac{\text{(}\text{Q}_{\text{e}}^{\text{2}}\text{×}\text{k}_{\text{2}}\text{×t)}}{\text{1+}\text{Q}_{\text{e}}\text{×t×}\text{k}_{\text{2}}}$ S4

Where *Q*_t_ (mg g^−1^) is the amount of analyte adsorbed at the given time t (min), *Q*_e_ (mg g^−1^) is the amount of PFOA adsorbed on the adsorbent after equilibrium. *k*_2_ (g mg^−1^ min^−1^) is the rate constant of adsorption, and the initial adsorption rate *h*_0_ (mg g^−1^ min^−1^) is defined as:

$\text{h}_{\text{0}}\text{=}\text{k}_{\text{2}}\text{*}\text{Q}_{\text{e}}^{\text{2}}$ S5

To quantify the temperature dependence, the activation energy *E*_a_ of the reaction was calculated from the slope of the Arrhenius plot (ln(*k*_2_) vs. *T*^−1^) according to:

$$\text{ln(}k_{2}\text{) = }\ln\left( \text{A} \right)-\frac{E_{a}}{\text{RT}}\text{ }\text{ }\text{ S6}$$

where *A* is the pre-exponential factor, *E*_a_ is the apparent activation energy (J mol^−1^), *R* is the gas constant (8.314 J mol^−1^ K^−1^), and *T* is the absolute temperature (K). A plot of ln (*k*_2_) vs. 1/*T* (or 1000/*T*) was fitted linearly, and *E*_a_ was obtained from the slope.

**PFOA removal experiment** was performed by adding FHCP-3 (3 mg) into the aqueous PFOA solution (100 mg L^−1^, 10 mL). After shaking for one day at room temperature, the supernatant after filtration was analysed by LC-MS. The removal efficiency of targeted PFOA was determined by:

$\text{Efficienc}\text{y }\left( \% \right)=100\times\frac{\text{C}_{\text{0 }}\text{-}\text{C}_{\text{t}}}{\text{C}_{\text{0}}}$ S7

Where *C*_0_ (mg L^−1^) and *C*_t_ (mg L^−1^) are the initial and residual concentrations of analytes in the original solution, respectively.

The adsorbed analytes were determined by:

$$\text{Q}_{\text{t}}\text{=}\frac{\text{(}\text{C}_{\text{0}}\text{-}\text{C}_{\text{t}}\text{)×}\text{V}_{\text{s}}}{\text{m}_{\text{a}}}\text{ }\text{ }\text{ }\text{ }\text{ }\text{S8}$$

Where *Q*_t_ (mg g^−1^) is the amount of analytes adsorbed on the FHCP at a given time t (min), *C*_0_ (mg L^−1^) is the initial concentration, *C*_t_ (mg L^−1^) is the concentration of analytes at given time t (min), *V*_s_ (L) is sample volume, and *m*_a_ (g) is the weight of added adsorbent.

**Regeneration of FHCP-3.** FHCP-3 was first saturated with PFOA by equilibrating 5 mg of sorbent in a 2000 mg L^−1^ PFOA solution, which corresponds to the maximum soluble concentration achievable under our experimental conditions and ensures saturation. After equilibrium was reached, desorption was performed using methanol at a solvent-to-sorbent ratio of 50 mL g^−1^ under continuous shaking at room temperature for 24 h, resulting in near-quantitative elution of PFOA. The desorbed material was thoroughly rinsed, dried, and reused in subsequent adsorption-desorption cycles to evaluate regeneration performance.

**Batch sorption experiments** were performed to evaluate the adsorption behaviour of FHCP-3 under environmentally relevant PFOA concentrations. A 100 µg L^−1^ PFOA working solution was prepared by diluting the stock solution with DI water, tap water (TW), or lake water (LW). For each experiment, 5 mg of FHCP-3 was added to 10 mL of PFOA solution in 25 mL polypropylene tubes and equilibrated at 25 ℃ under continuous shaking for 24 h. To examine the effects of water chemistry, experiments were conducted in DI, TW, and LW at their native pH, as well as in acidified matrices (pH 2), which were adjusted by dropwise addition of 0.1 mol L^−1^ HCl. Ionic strength effects were assessed by adding NaCl at 0, 1, 10, and 100 mmol L^−1^. Competitive ion studies were conducted by adding representative monovalent (Na^+^, K^+^) and divalent (Ca^2+^, Mg^2+^) cations, as well as Cl^−^, NO_3_^−^, and SO_4_^2−^ anions at 1 mmol L^−1^. Natural organic matter interference was evaluated using humic acid (HA) at 5 mg L^−1^. All experiments were carried out in triplicate, and equilibrium concentrations were determined using LC-MS.

**Calculation of *K*_D_ and log*K*_D_**. The solid-liquid distribution coefficient (*K*_D_) was calculated to quantify the intrinsic sorption affinity of FHCP-3 toward PFOA at environmentally relevant concentrations. *K*_D_ was determined using the following equation:

$$\text{K}_{\text{D}}\text{=}\frac{\left( \text{C}_{\text{0}}\text{-}\text{C}_{\text{e}} \right)\text{×}\text{V}}{\text{C}_{\text{e}}\text{×}\text{m}} \text{ S9}$$

where *C*_0_ and *C*_e_ are the initial and equilibrium concentrations of PFOA (µg L^−1^), V is the volume of the PFOA solution (L), m is the mass of the sorbent (g), and *K*_D_ is the distribution coefficient (L g^−1^).

The logarithmic form, log*K*_D_, was obtained as:

$$\log\text{K}_{\text{D}}\text{=}\log\text{(}\frac{\text{C}_{\text{0}}\text{-}\text{C}_{\text{e}}}{\text{C}_{\text{e}}}\text{×}\frac{\text{V}}{\text{m}}\text{) }\text{ }\text{ }\text{ }\text{ }\text{ }\text{ S10}$$

*K*_D_ and log*K*_D_ provide normalised measures of sorption affinity that account for both sorbent dosage and equilibrium concentration, making them particularly suitable for evaluating PFOA adsorption at trace levels. These metrics are widely used in PFOA sorption studies to compare affinity across different matrices and to assess robustness under realistic water chemistries.

## 1.2. Theoretical calculation

All quantum chemical calculations were conducted using the Gaussian 16 software package.^[1]^ Geometry optimizations and vibrational frequency analyses were performed at the B3LYP/6-31G (d, p) level of theory with Grimme’s D3 dispersion correction.^[2-4]^ Solvent effects were included via the SMD implicit solvation model for water. To obtain more accurate electronic energies, single-point energy calculations were carried out at the B3LYP/def2-TZVP level, with counterpoise correction applied to eliminate basis set superposition error.^[5]^ The binding energy (*∆E*) was computed using the following equation:

$$\Delta E=E_{complex}-E_{polymer}-E_{PFOA}$$

Here, *E_complex_*, *E_polymer_*, and *E_PFOA_* represent the electronic energies of the optimized FHCP-PFOA complex, the isolated polymer fragment, and the isolated PFOA molecule, respectively. Two distinct binding configurations (FHCP-PFOA-1 and FHCP-PFOA-2) were considered to evaluate the binding strength of PFOA to the fluorinated polymer. A more negative *∆E* indicates a stronger non-covalent interaction in the gas phase.

To better reflect the thermodynamic feasibility of the adsorption process, Gibbs free energies (*∆G*) were also estimated. For the solvated state, B3LYP/6-311G (d) and M05-2X/6-31G (d) levels were employed. Thermodynamic corrections and partition functions were obtained using the Shermo program^[6]^. The *∆G* was calculated as:

$$\Delta G=G_{complex}-G_{polymer}-G_{PFOA}$$

Where *complex* refers to either the FHCP-PFOA-1 or FHCP-PFOA-2 complex, representing two distinct binding configurations between the polymer and PFOA. The *∆G* were computed separately for each complex to assess the thermodynamic feasibility of both binding modes.

Electrostatic potential (ESP) analysis was performed using Multiwfn 3.8,^[7]^ and the visualisations were generated with VMD 1.9.3.^[8]^ To visualise and identify noncovalent interactions between FHCP and PFOA, reduced density gradient (RDG) analysis was performed based on the optimised DFT structures. The wavefunction file was generated at the B3LYP/6-31G (d, p) level using Gaussian 16. The RDG isosurfaces and the corresponding sign (λ_2_) ρ vs. RDG scatter plots were analysed and visualised using the Multiwfn 3.8 software and visualised with VMD. In the RDG plot, green regions correspond to van der Waals interactions, while blue and red regions indicate strong attractive (e.g., hydrogen bonding) and repulsive (steric) interactions, respectively.

# 2. Figures (S1-S27)

## 2.1 Synthetic routes to FHCPs

**Figure S1.** Schematic illustration of fluorine-containing hypercrosslinked polymer synthesis.

## 2.2. Structural characterisation of HCPs and FHCPs

**Figure S2.** XPS high-resolution (a) C 1s and (b) F 1s spectra of HCP and FHCPs.

## 2.3. Adsorption performance

**Figure S3.** Chemical structure and size of PFOA.

**Figure S4.** Calibration curve for PFOA concentration measurement using LC-MS. In the measured range, there is a good linear association between the peak area and the PFOA concentration (R^2^ = 0.9996).

**Figure S5.** Equilibrium PFOA adsorption capacity, *Q*_e_, of (a) FHCP-1 and (b) FHCP-2, as a function of equilibrium PFOA concentration, *C*_e_, fitted with Langmuir (red line) and Freundlich models (blue line).

**Figure S6.** Sorption kinetics of PFOA (*C*_0_ = 1 mg L^−1^) onto FHCP-3, fitted with a pseudo-first-order model.

**Figure S7.** Sorption kinetics of PFOA (*C*_0_=1 mg L^−1^) onto FHCP-3, fitted with a pseudo-second-order model.


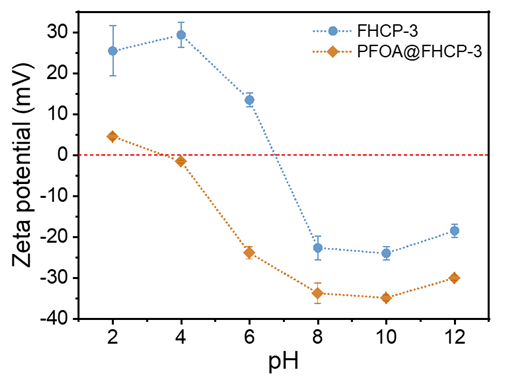


**Figure S8.** Zeta potential of pristine FHCP-3 and after adsorption of PFOA.


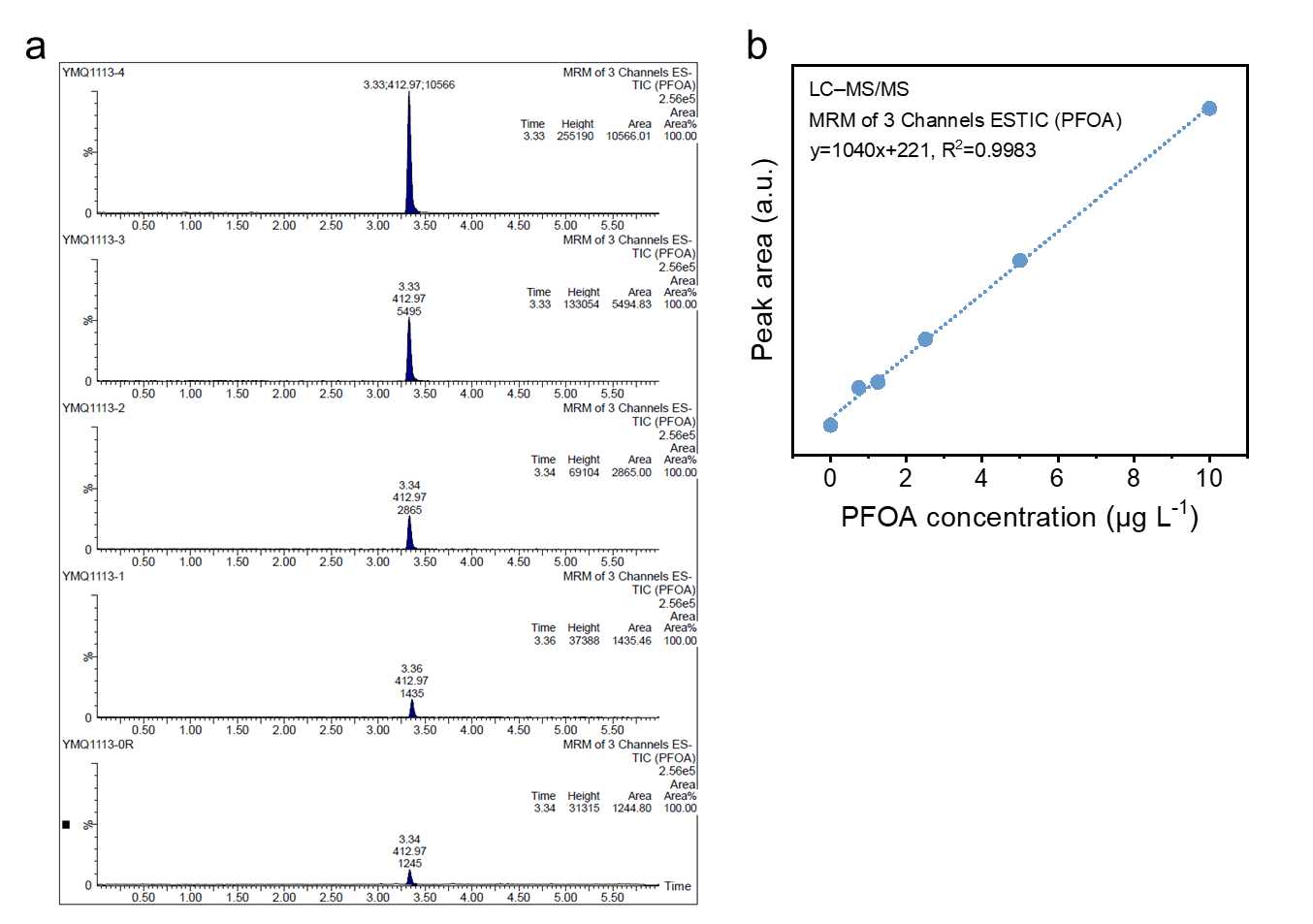


**Figure S9.** (a) Raw LC-MS chromatograms of PFOA standard solutions (0.75-10 µg L^−1^) acquired on a Waters Xevo TQ-S Cronos triple-quadrupole mass spectrometer in MRM mode. (b) Calibration curve constructed from the peak areas of the standards (R^2^=0.9983), demonstrating excellent linearity over the concentration range used for quantification.


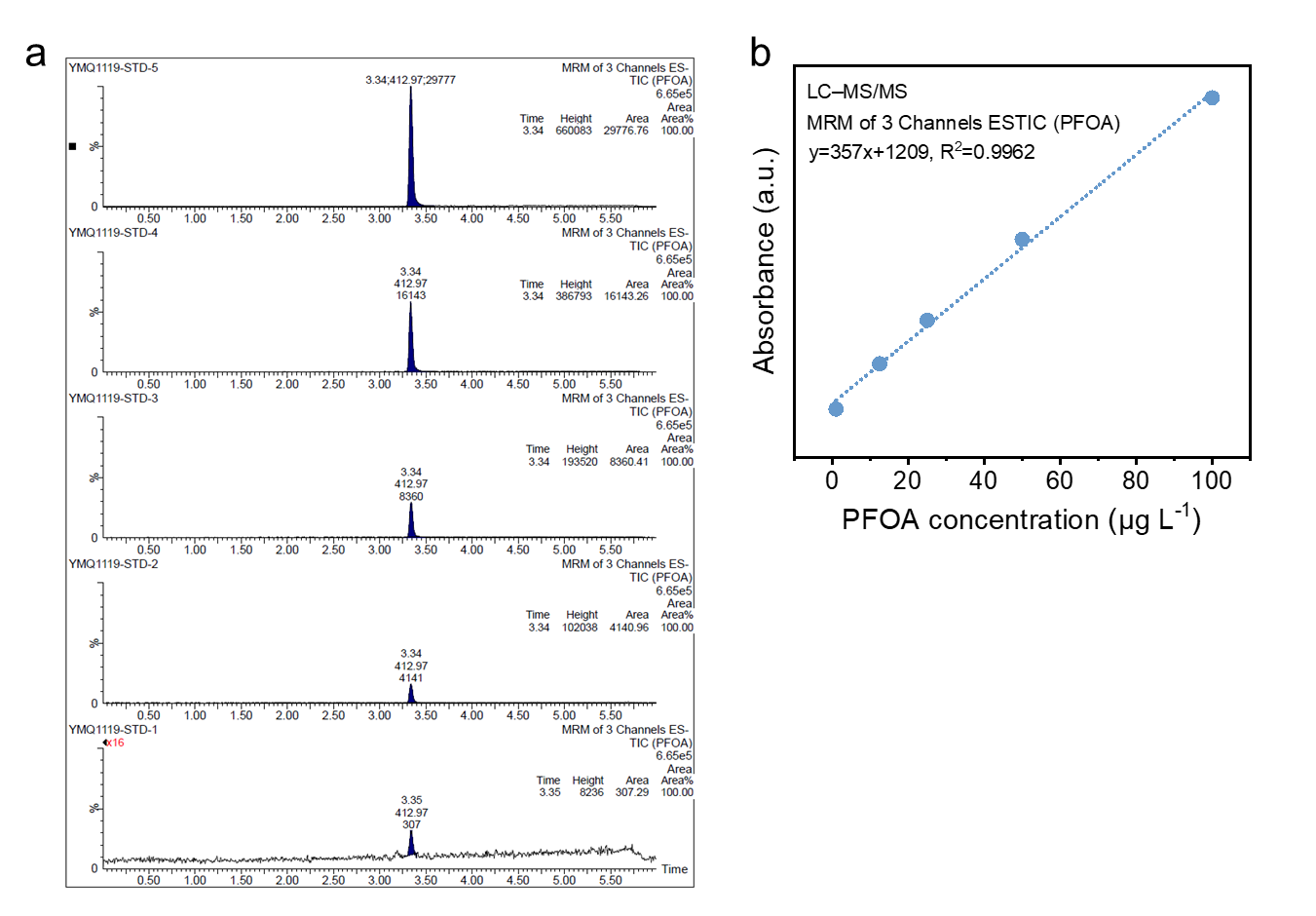


**Figure S10.** (a) Raw LC-MS chromatograms of PFOA standard solutions (1-100 µg L^−1^) acquired on a Waters Xevo TQ-S Cronos triple-quadrupole mass spectrometer in MRM mode. (b) Calibration curve constructed from the peak areas of the standards (R^2^=0.9962), demonstrating excellent linearity over the concentration range used for quantification.


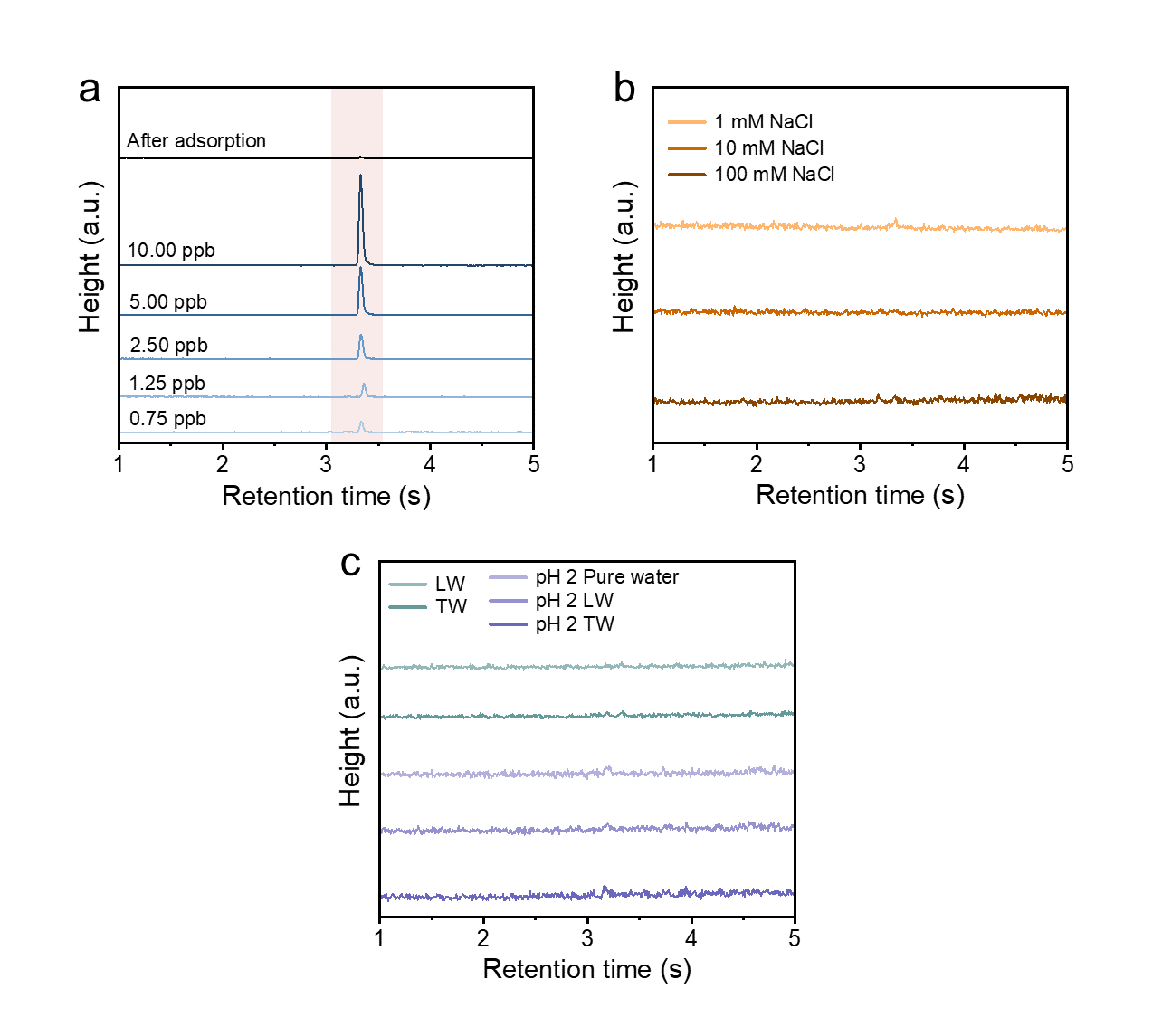


**Figure S11.** (a) LC-MS chromatograms before and after adsorption at 10 ppb PFOA. (b) LC-MS chromatograms of post-adsorption PFOA solutions (initial concentration 10 ppb) in the presence of different NaCl concentrations. (c) LC-MS chromatograms of post-adsorption PFOA solutions (initial concentration 10 ppb) in different water matrices and at acidic pH.


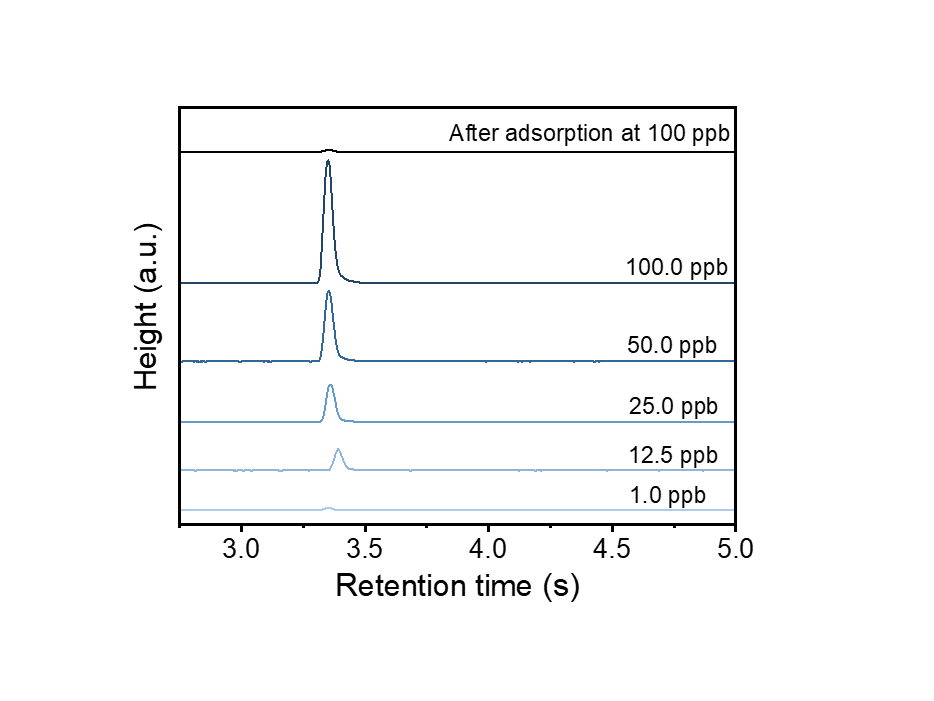


**Figure S12.** LC-MS chromatograms of PFOA standard solutions (1.0-100.0 µg L^−1^) and the residual PFOA concentration after adsorption by FHCP-3 from a 100 µg L^−1^ solution.


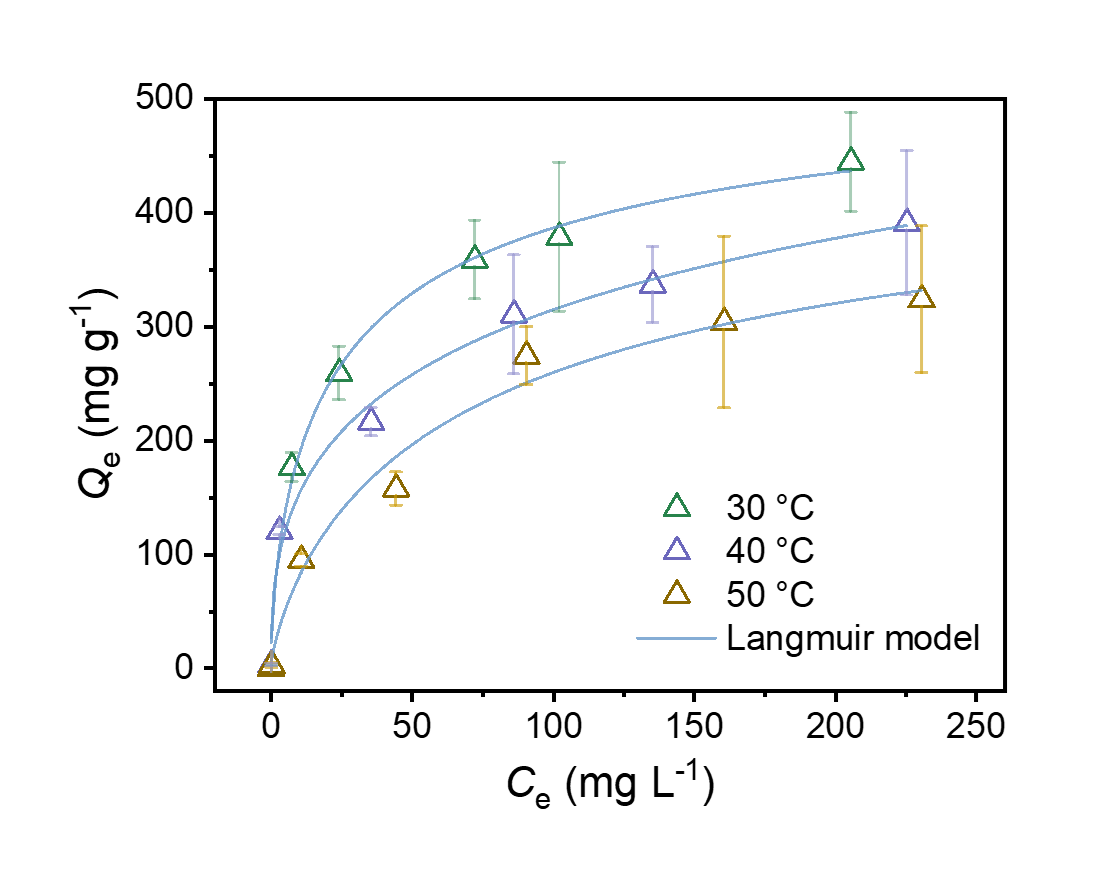


**Figure S13.** Temperature-dependent adsorption isotherms of PFOA on FHCP-3. Equilibrium adsorption isotherms measured at 30, 40, and 50 ℃ after 24 h of contact time. Error bars indicate the standard deviation from three independent replicates (n=3). Solid lines represent Langmuir model fits. The equilibrium adsorption capacity generally decreased with increasing temperature, indicating that elevated temperature is unfavorable for equilibrium uptake. Although the Langmuir model captured the overall trend, larger deviations were observed at higher equilibrium concentrations.


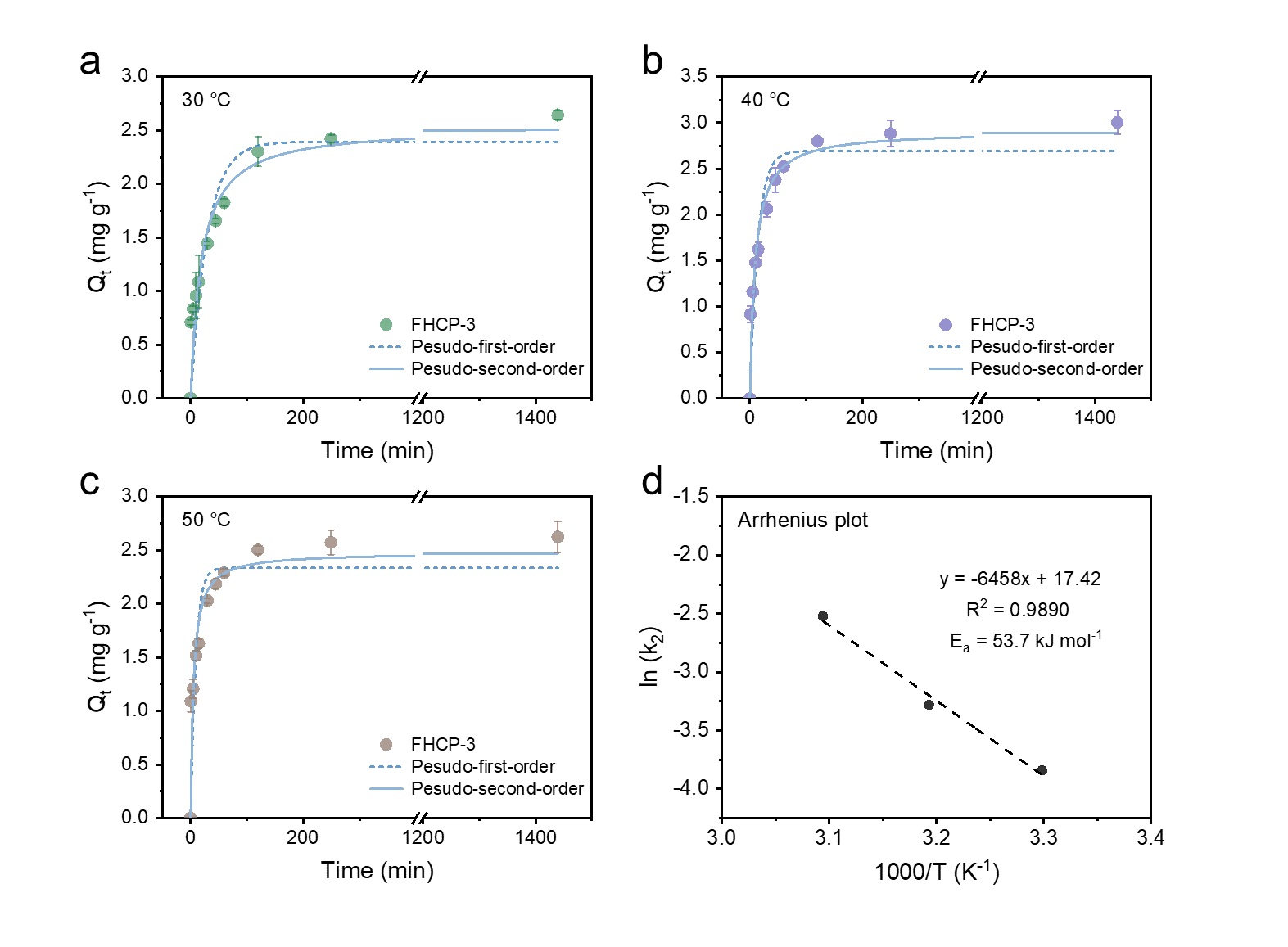


**Figure S14.** Temperature-dependent adsorption kinetics and Arrhenius analysis. (a-c) Time-dependent uptake (*Q*_t_) of PFOA (*C*_0_=1 mg L^−1^) on FHCP-3 at 30, 40, and 50 ℃, respectively. Experimental data are fitted using the pseudo-first-order and pseudo-second-order kinetic models. (d) Arrhenius plot of ln (*k*_2_) versus 1000/*T* derived from the pseudo-second-order rate constants, yielding an apparent activation energy *E*_a_ of 53.7 kJ mol^−1^.


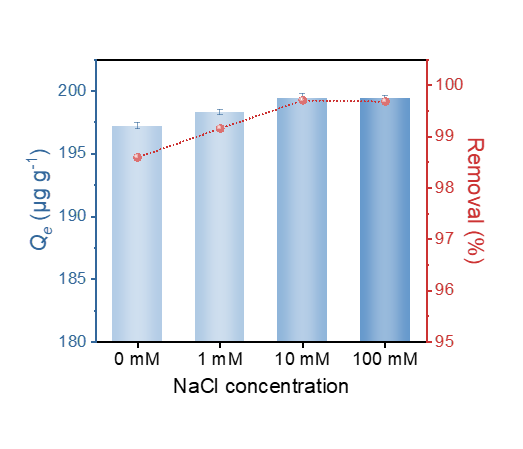


**Figure S15.** Adsorption capacities and efficiencies of FHCP-3 under varying ionic strengths. Deionised water was used as matrix, *C*_0_=100 µg L^−1^, sorbent dosage=0.5 g L^−1^, reaction time was 24 h, solution volume was 10 mL, and solution pH was ~6.5. Error bars represent the standard deviation of three repeated experiments, and central values denote data averages.

## 2.4. Adsorbent recovery and regeneration


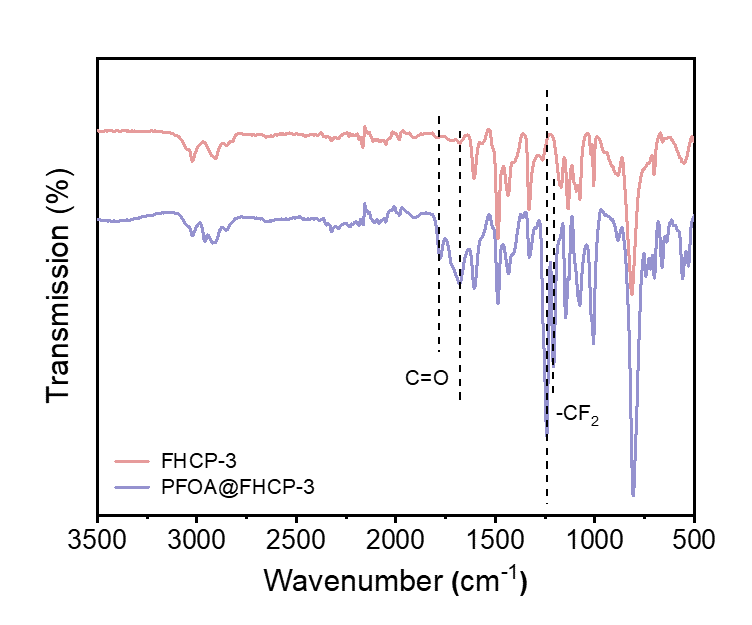


**Figure S16.** FTIR spectra of FHCP-3 and PFOA@FHCP-3.

**Figure S17.** XPS survey spectra of FHCP-3 and PFOA@FHCP-3.

**Figure S18.** N_2_ adsorption-desorption isotherms and pore size distributions of FHCP-3 and PFOA@FHCP-3.


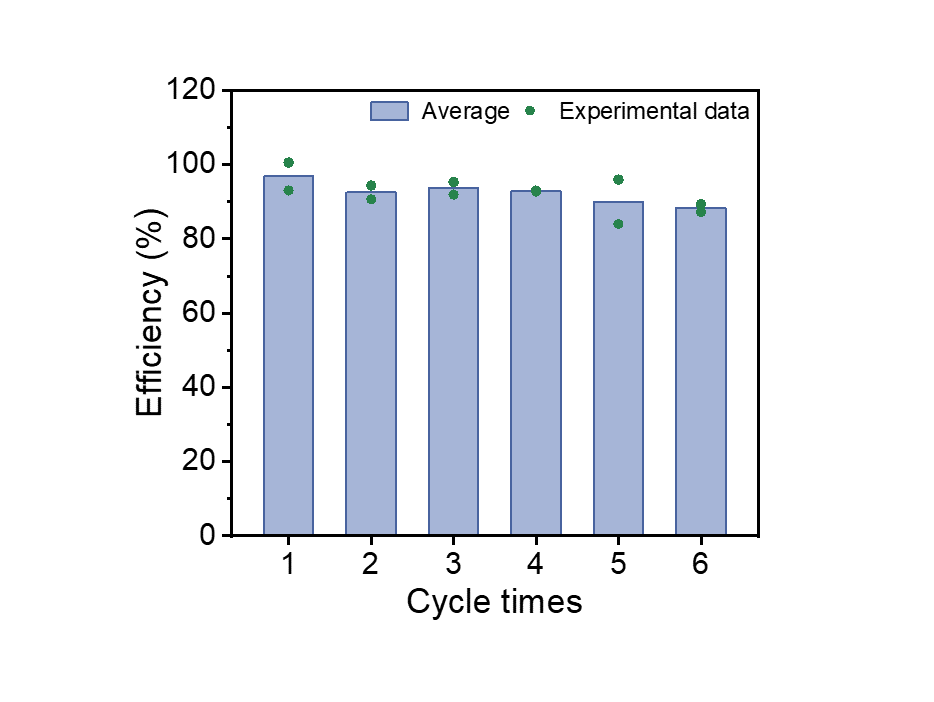


**Figure S19.** PFOA adsorption efficiency of FHCP-3 over six successive adsorption and regeneration cycles using a PFOA *C*_0_ of 100 ppm.

**Figure S20.** FTIR of PFOA@FHCP-3 and regenerated FHCP-3.

**Figure S21.** N_2_ sorption-desorption isotherms and pore size distribution of FHCP-3 and regenerated FHCP-3. The regenerated sample shows a slight decrease in surface area (from 1689 to 1613 m^2^ g^−1^), while maintaining a similar pore size distribution, indicating good structural stability.


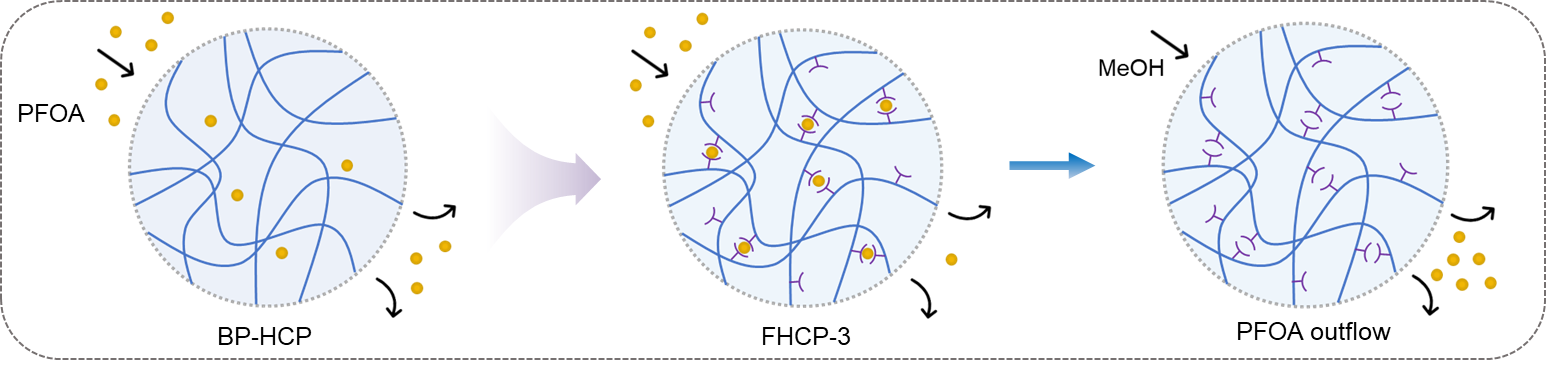


**Figure S22.** Schematic comparison of the adsorption and desorption process.


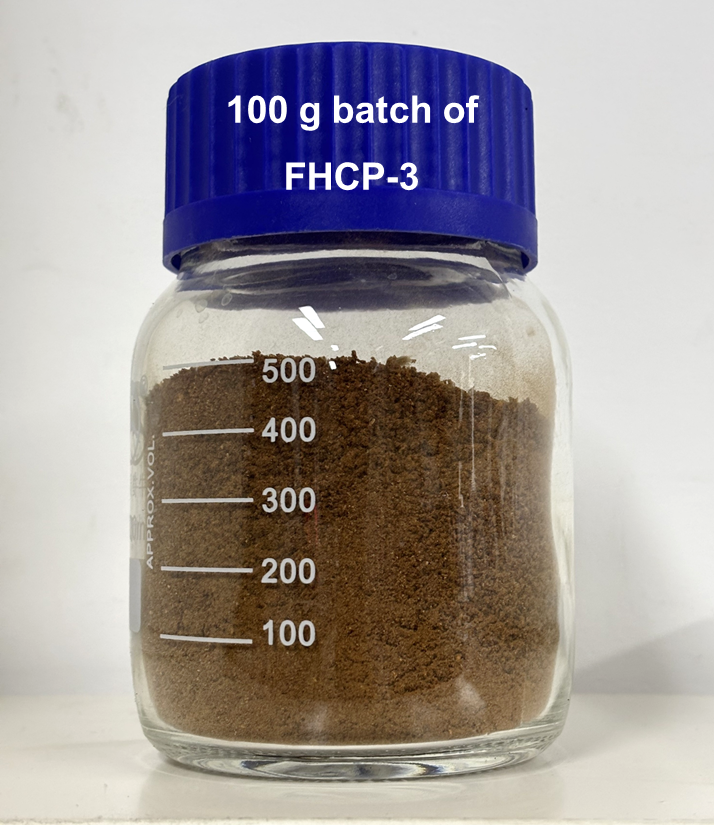


**Figure S23.** Photograph of FHCP-3 obtained from repeated scaled-up syntheses. Approximately 100 g of FHCP-3 was accumulated as a brown powder through multiple preparative runs using enlarged reaction vessels under laboratory conditions.

## 2.5. Mechanistic insight


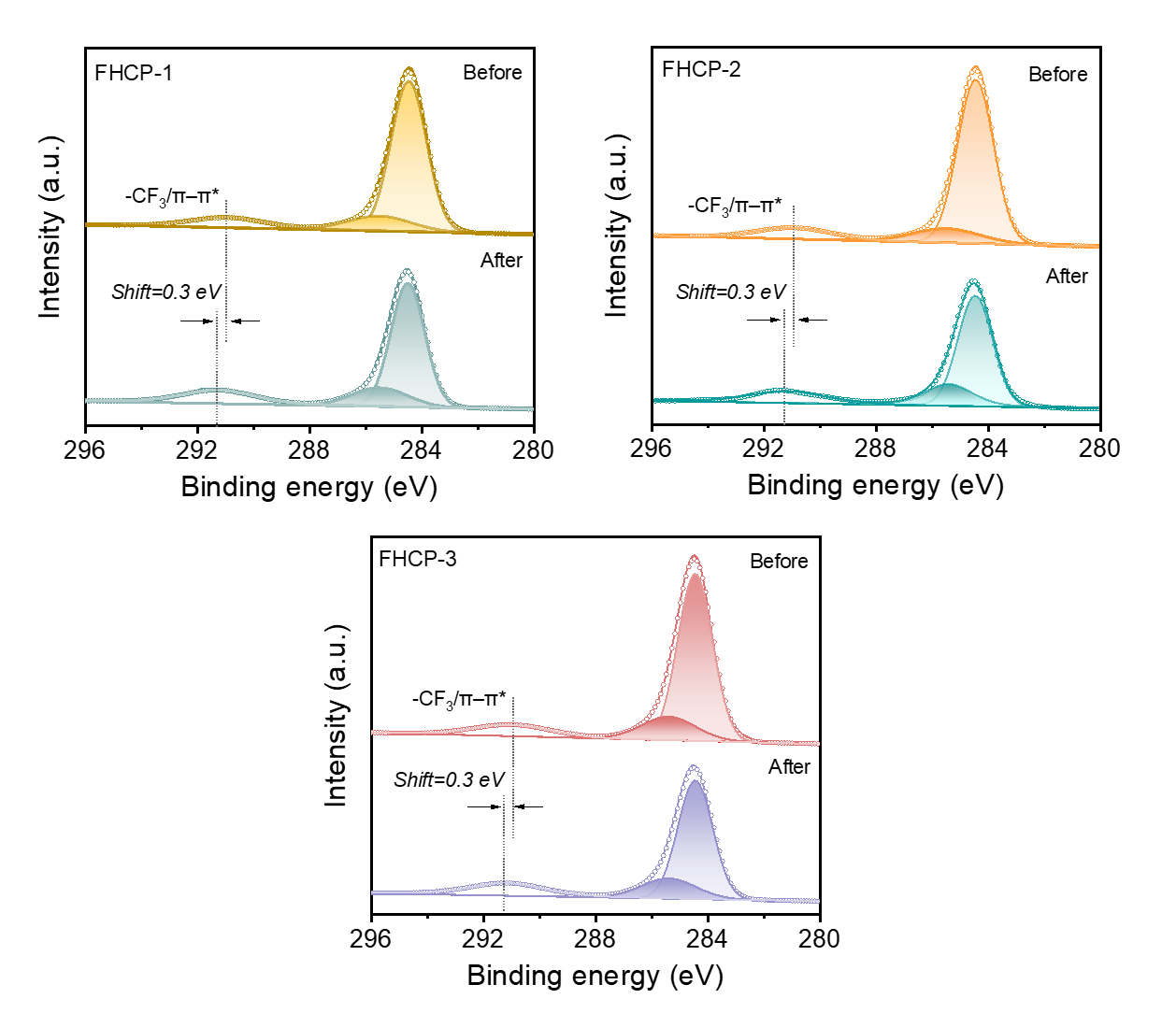


**Figure S24.** High-resolution XPS C1s spectra of FHCP-1, FHCP-2, and FHCP-3, along with PFOA-preloaded fluorinated polymers. Dotted lines indicate the overlapping region of -CF_3_ and π-π* transitions. Binding energy shifts of 0.3 eV were observed after PFOA adsorption.


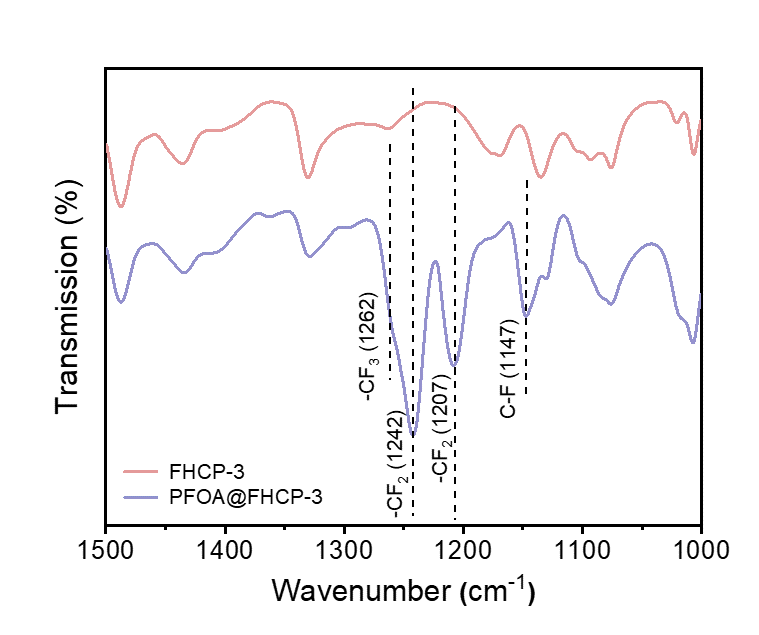


**Figure S25.** FTIR spectra of FHCP-3, along with PFOA-loaded fluorinated polymers.


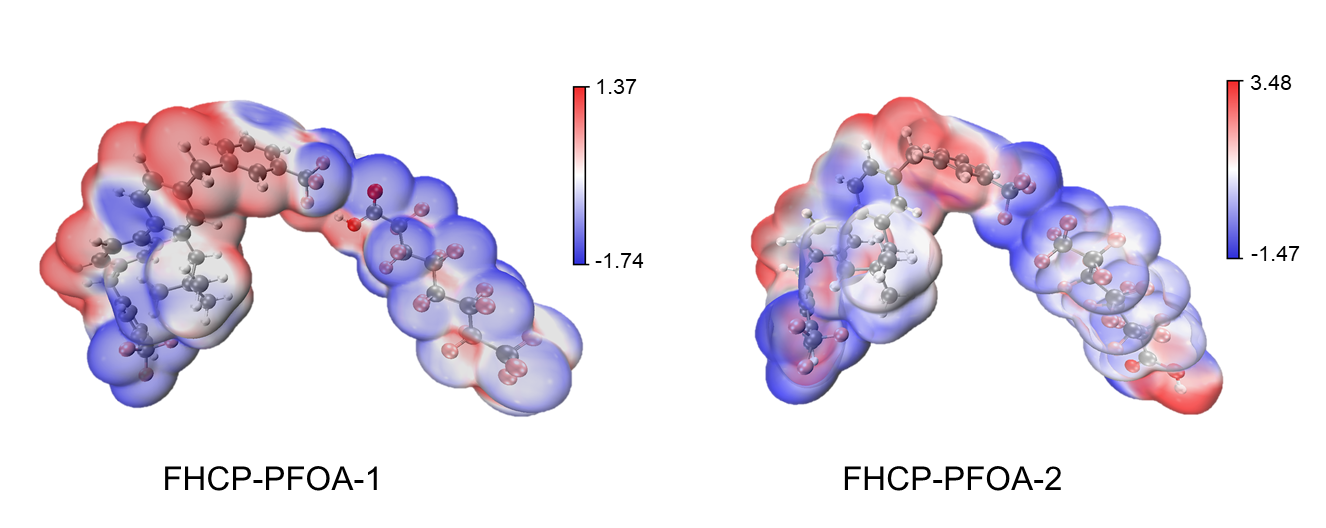


**Figure S26.** Intermolecular vdW surface penetration maps with ESP colouring of FHCP-1 (hydrogen bonding), and FHCP-PFOA-2 (fluorophilic interaction).


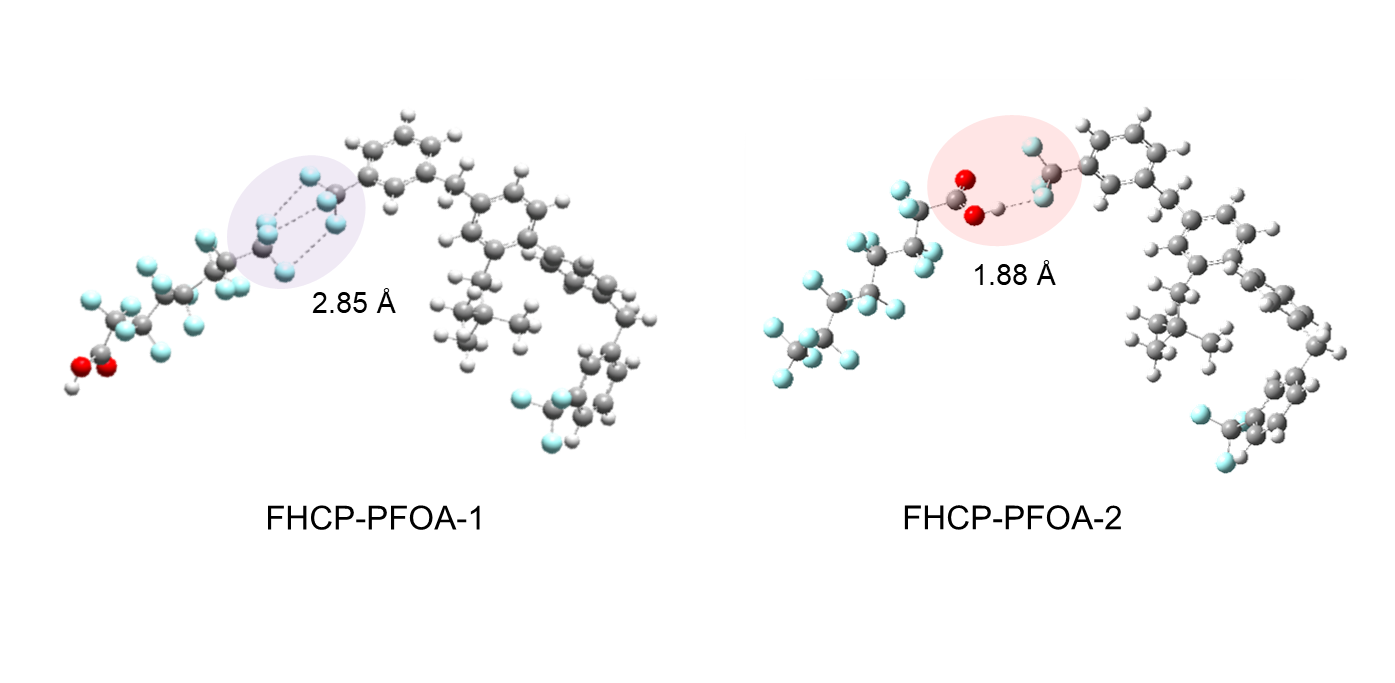


**Figure S27.** Optimised molecular structure of FHCP-PFOA-1 and FHCP-PFOA-2.

# 3. Tables (S1-S7)

**Table S1.** Composition of (F)HCPs in weight (%) determined by XPS.

|  | C (%) | O (%) | Cl (%) | F (%) |
| --- | --- | --- | --- | --- |
| BP-HCP | 92.98 | 3.56 | 3.46 | 0.00 |
| FHCP-1 | 90.99 | 3.25 | 4.27 | 1.49 |
| FHCP-2 | 91.33 | 3.52 | 3.40 | 1.75 |
| FHCP-3 | 91.58 | 3.21 | 2.85 | 2.85 |

**Table S2.** Porous properties of (F)HCPs, including BET specific surface area (SSA_BET_), micropore volume (V_Micro_), mesopore volume (V_Meso_), and total pore volume (V_Total_). Determined via QS-DFT on the adsorption branch of N_2_ isotherms, collected at 77 K.

|  | SSA_BET_  (m^2^ g^-1^) | V_Micro_  (cm^3^ g^-1^) | V_Meso_  (cm^3^ g^-1^) | V_Total_  (cm^3^ g^-1^) | V_Micro_/V_Total_  (%) |
| --- | --- | --- | --- | --- | --- |
| BP-HCP | 1769 ± 79 | 1.58 | 1.49 | 3.08 | 51 |
| FHCP-1 | 1591 ± 99 | 1.62 | 1.10 | 2.72 | 60 |
| FHCP-2 | 1697 ± 51 | 1.36 | 1.28 | 2.64 | 52 |
| FHCP-3 | 1740 ± 61 | 1.34 | 1.67 | 3.01 | 45 |
| PFOA@  FHCP-3 | 1490 ± 93 | 0.88 | 1.16 | 2.04 | 43 |

**Table S3.** Fitting results and correlation coefficients of the Langmuir and Freundlich models for the adsorption performance of (F)HCPs. Maximum adsorption capacity, *Q*_e_, and theoretical maximum adsorption capacity, *Q*_m_, are provided for each network.

|  |  | | Langmuir model | | | Freundlich model | | | | |
| --- | --- | --- | --- | --- | --- | --- | --- | --- | --- | --- |
|  | *Q*_e_  /mg g^−1^ | *Q_m_*  /mg g^−1^ | | *K_L_*  /L mg^−1^ | *R^2^* | | *K_F_*  (mg g^−1^) (L mg^−1^)^1/n^ | 1/n | *R^2^* |  |
| BP-HCP | 867 ± 150 | 1234 | | 0.0014 | 0.997 | | 13.4 | 0.57 | 0.974 |  |
| FHCP-1 | 968 ±110 | 1305 | | 0.0020 | 0.983 | | 23.9 | 0.51 | 0.929 |  |
| FHCP-2 | 1246 ± 120 | 1542 | | 0.0023 | 0.994 | | 35.1 | 0.49 | 0.974 |  |
| FHCP-3 | 1550 ± 60 | 1956 | | 0.0022 | 0.992 | | 34.5 | 0.53 | 0.968 |  |

**Table S4.** PFOA adsorption capacity of a variety of reported adsorbents.

| Adsorbent | Adsorbent category | *Q*_m_ (mg g^−1^) | Reference |
| --- | --- | --- | --- |
| PEF-f-CMC | Cellulose | 2.32 | ^[9]^ |
| Poly-SOMS | Silica | 10 | ^[10]^ |
| DFB-CDP | Crosslinked supermolecule | 34 | ^[11]^ |
| DFB-CDP-3 |  | 65 | ^[12]^ |
| DFB-CDP-2 |  | 68.2 |  |
| DFB-CDP-1 |  | 86.6 |  |
| P2-9+@IONPs | Fluoropolymer | 219 | ^[13]^ |
| FSQ-1 | Covalent-Organic Framework (COF) | 370 | ^[14]^ |
| All-silica Beta | Zeolite | 350 | ^[15]^ |
| UiO-66-F4 | Metal-Organic Framework (MOF) | 467 | ^[16]^ |
| NU-1000 |  | 507 | ^[17]^ |
| PCN-999 |  | 1089 | ^[18]^ |
| FHCP-3 | Hypercrosslinked polymer (HCP) | 1956 | This work |
| PAF-1-NDMD | Porous organic framework (POF) | 2000 | ^[19]^ |
| TG-PD COF | COF | 2600 | ^[20]^ |

**Table S5.** Parameters obtained by fitting the experimental data of the adsorption of PFOA on FHCP-3 to pseudo-first-order and pseudo-second-order models.

| Pseudo-first-order-kinetic model | | | | |  | Pseudo-second-order-kinetic model | | | | |
| --- | --- | --- | --- | --- | --- | --- | --- | --- | --- | --- |
| *T*  (℃) | *Q*_e_  (mg g^−1^) | *Q*_m_  (mg g^−1^) | *k*_1_  (min^−1^) | *R*^2^ |  | *Q*_m_  (mg g^−1^) | *k_2_*  (g mg^−1^ min^−1^) | *h*_0_  (mg g^−1^ min^−1^) | | *R*^2^ |
| 30 | 2.4±0.2 | 2.4 | 0.034 | 0.851 |  | 2.54 | 0.021 | | 0.135 | 0.916 |
| 40 | 2.5±0.5 | 2.7 | 0.072 | 0.879 |  | 2.91 | 0.038 | | 0.322 | 0.939 |
| 50 | 2.5±0.2 | 2.3 | 0.114 | 0.823 |  | 2.48 | 0.080 | | 0.492 | 0.900 |

**Table S6.** *K*_D_ and log*K*_D_ values of FHCP-3 for PFOA (100 ppb) in various water matrices.

| Matrix | *C*_0_ (µg L^−1^) | *C*_e_ (µg L^−1^) | *K*_D_ (L g^−1^) | log*K*_D_ |
| --- | --- | --- | --- | --- |
| DI water | 100 | 0.33* | 600 | 2.78 |
| Tap water (TW) | 100 | 0.41* | 491 | 2.69 |
| Lake water (LW) | 100 | 0.65 | 306 | 2.49 |
| pH 2, DI blank | 100 | 0.26* | 779 | 2.89 |
| pH 2, TW | 100 | 0.60 | 329 | 2.52 |
| pH 2, LW | 100 | 0.63 | 315 | 2.50 |

*Values near the limit of quantification (LOQ) and should be considered semi-quantitative.

**Table S7.** Estimated cost price per gram of FHCP-3 and representative PFAS sorbents used in water treatment. A description of cost estimation procedures is provided below.

| PFOA adsorbent | Estimated cost (€ g^−1^) | Source |
| --- | --- | --- |
| BP-HCP | 1.52 | This work |
| FHCP-1 | 2.24 | This work |
| FHCP-2 | 0.91 | This work |
| FHCP-3 | 0.52 | This work |
| Activated carbon | 0.02-0.10 | Commercial |
| S-8 Macroporous Adsorption Resin | 0.17 | Commercial |
| Ion Exchange Resin 12627-85-9 | 1.53 | Commercial |
| Anion Exchange Resin Dowex | 1.70 | Commercial |
| Ion Exchange Resin CG-50 | 3.87 | Commercial |
| QFgel | 9.39 | Literature^[21]^ |
| Chitosan/F-COF | 13 | Literature^[22]^ |
| F-MOF | 47 | Literature^[23]^ |
| PAF-1-NDMB | 72.12 | Literature^[19]^ |
| FSQ-1 | 120 | Literature^[14]^ |

Prices sourced from approved laboratory suppliers listed in the Donghua University procurement platform (accessed in December 2025).

**Cost estimation**

Estimated cost per gram of FHCPs for PFOA adsorption was calculated and compared with representative commercial sorbents and previously reported COF/MOF adsorbents in Table S7. Estimated material costs were determined based on the reagents required for the synthesis of each polymer. The unit prices were obtained from certified laboratory suppliers listed in the Donghua University procurement platform, using the lowest available unit price for 100 g (solids) or 1 L (liquids) and adjusted according to the stoichiometric quantities used in the reaction. A minimum reagent purity of ≥98% was applied. When this specification was not available, the closest alternative meeting the purity requirement was selected. Work-up steps such as washing or Soxhlet extraction were excluded from the calculation, and labour, synthesis time, equipment depreciation, and energy consumption were likewise not considered. We emphasise that the calculated values are intended solely for comparative assessment and reflect lab-scale reagent pricing rather than industrial production costs.

# 4. Supplementary References

[1] M. J. Frisch, G. W. Trucks, H. B. Schlegel, et al., Wallingford, CT 2016.

[2] Becke, D. Axel, "Density‐Functional Thermochemistry. III. The Role of Exact Exchange," *Journal of Chemical Physics* (1993): 98, 7, 5648.

[3] P. C. P. Hariharan, J. A. Pople, "The Influence of Polarization Functions on Molecular Orbital Hydrogenation Energies," *Theoretica chimica acta* (1973): 28, 213.

[4] S. Grimme, J. Antony, S. Ehrlich, H. Krieg, "A Consistent and Accurate ab initio Parametrization of Density Functional Dispersion Correction (DFT-D) for the 94 Elements H-Pu," *Journal of Chemical Physics* (2010): 132, 15, 154104.

[5] F. Weigend, R. Ahlrichs, "Balanced Basis Sets of Split Valence, Triple Zeta Valence and Quadruple Zeta Valence Quality for H to Rn: Design and Assessment of Accuracy," *Physical Chemistry Chemical Physics Pccp* (2005): 7, 18, 3297.

[6] T. Lu, Q. Chen, "Shermo: A General Code for Calculating Molecular Thermochemistry Properties," *Computational and Theoretical Chemistry* (2021), 113249.

[7] T. Lu, F. Chen, "Multiwfn: A Multifunctional Wavefunction Analyzer," *Journal of Computational Chemistry* (2012): 33, 5, 580.

[8] W. Humphrey, A. Dalke, K. Schulten, "VMD: Visual Molecular Dynamics,"*J Mol Graph* (1996): 14, 1, 33.

[9] M. Ateia, M. F. Attia, A. Maroli, et al., "Rapid removal of poly-and perfluorinated alkyl substances by poly (ethylenimine)-functionalized cellulose microcrystals at environmentally relevant conditions," *Environmental Science & Technology Letters* (2018): 5, 12, 764.

[10] E. K. Stebel, K. A. Pike, H. Nguyen, et al., "Absorption of Short-Chain to Long-Chain Perfluoroalkyl Substances Using Swellable Organically Modified Silica," *Environmental Science: Water Research & Technology* (2019): 5, 11, 1854.

[11] L. Xiao, Y. Ling, A. Alsbaiee, C. Li, D. E. Helbling, W. R. Dichtel, "β-Cyclodextrin Polymer Network Sequesters Perfluorooctanoic Acid at Environmentally Relevant Concentrations," *Journal of the American Chemical Society* (2017): 139, 23, 7689.

[12] L. Xiao, C. Ching, Y. Ling, et al., "Cross-Linker Chemistry Determines the Uptake Potential of Perfluorinated Alkyl Substances by β-Cyclodextrin Polymers," *Macromolecules* (2019): 52, 10, 3747.

[13] X. Tan, P. Dewapriya, P. Prasad, et al., "Efficient Removal of Perfluorinated Chemicals from Contaminated Water Sources using Magnetic Fluorinated Polymer Sorbents," *Angewandte Chemie International Edition* (2022): 61, 49, e202213071.

[14] J. Huang, Y. Shi, G. Z. Huang, et al., "Facile Synthesis of a Fluorinated‐Squaramide Covalent Organic Framework for the Highly Efficient and Broad‐Spectrum Removal of Per‐and Polyfluoroalkyl Pollutants," *Angewandte Chemie International Edition* (2022): 61, 31, e202206749.

[15] M. Van Den Bergh, A. Krajnc, S. Voorspoels, et al., "Highly selective removal of perfluorinated contaminants by adsorption on all‐silica zeolite beta," *Angewandte Chemie* (2020): 132, 33, 14190.

[16] K. Sini, D. Bourgeois, M. Idouhar, M. Carboni, D. Meyer, "Metal–organic framework sorbents for the removal of perfluorinated compounds in an aqueous environment," *New Journal of Chemistry* (2018): 42, 22, 17889.

[17] R. Li, S. Alomari, R. Stanton, et al., "Efficient Removal of Per-and Polyfluoroalkyl Substances from Water with Zirconium-Based Metal–Organic Frameworks," *Chemistry of Materials* (2021): 33, 9, 3276.

[18] R.-R. Liang, S. Xu, Z. Han, et al., "Exceptionally High Perfluorooctanoic Acid Uptake in Water by a Zirconium-Based Metal–Organic Framework through Synergistic Chemical and Physical Adsorption," *Journal of the American Chemical Society* (2024): 146, 14, 9811.

[19] X. Liu, C. Zhu, J. Yin, et al., "Installation of Synergistic Binding Sites onto Porous Organic Polymers for Efficient Removal of Perfluorooctanoic Acid," *Nature Communications* (2022): 13, 1, 2132.

[20] A. Jrad, G. Das, N. Alkhatib, et al., "Cationic Covalent Organic Framework for the Fluorescent Sensing and Cooperative Adsorption of Perfluorooctanoic Acid," *Nature Communications* (2024): 15, 1, 1.

[21] K. Fu, F. Luo, Z. Fang, et al., "Amphipathic Fluoroamine-Functionalized Hydrogels for Enhanced Selective Removal of Anionic PFAS from Water," *Nature Communications* (2025): 16, 1, 10152.

[22] C. He, Y. Yang, Y.-J. Hou, T. Luan, J. Deng, "Chitosan-coated fluoro-functionalized covalent organic framework as adsorbent for efficient removal of per- and polyfluoroalkyl substances from water," *Separation and Purification Technology* (2022): 294.

[23] S.-Y. Ma, J. Wang, L. Fan, H.-L. Duan, Z.-Q. Zhang, "Preparation of a Fluorinated Metal-Organic Framework and Its Application for the Dispersive Solid-Phase Extraction of Perfluorooctanoic Acid," *Journal of Chromatography A* (2020): 1611, 460616.
